# Up‐regulation of PRKDC was associated with poor renal dysfunction after renal transplantation: A multi‐centre analysis

**DOI:** 10.1111/jcmm.17737

**Published:** 2023-04-01

**Authors:** Zhijun Cao, Hao Jiang, Chunchun Zhao, Huifeng Zhou, Zheng Ma, Chen Xu, Jianglei Zhang, Minjun Jiang, Zhenfan Wang

**Affiliations:** ^1^ Department of Urology, Suzhou Ninth People's Hospital Soochow University Suzhou 215000 China; ^2^ Department of Urology The First Affiliated Hospital of Soochow University Suzhou 215000 China; ^3^ Department of Urology, Suzhou Municipal Hospital Nanjing Medical University Suzhou 215000 China; ^4^ Department of Haematology The Children's Hospital of Soochow University Suzhou 215000 China

**Keywords:** bioinformatic analysis, external validation, kidney transplantation, PRKDC, renal dysfunction

## Abstract

Renal transplantation is the only efficacious treatment for end‐stage kidney disease. However, some people have developed renal insufficiency after transplantation, the mechanisms of which have not been well clarified. Previous studies have focused on patient factors, while the effect of gene expression in the donor kidney on post‐transplant renal function has been less studied. Donor kidney clinical data and mRNA expression status were extracted from the GEO database (GSE147451). Weight gene co‐expression network analysis (WGCNA) and differential gene enrichment analysis were performed. For external validation, we collected data from 122 patients who accepted renal transplantation at several hospitals and measured the level of target genes by qPCR. This study included 192 patients from the GEO data set, and 13 co‐expressed genes were confirmed by WGCNA and differential gene enrichment analysis. Then, the PPI network contained 17 edges as well as 12 nodes, and four central genes (PRKDC, RFC5, RFC3 and RBM14) were identified. We found by collecting data from 122 patients who underwent renal transplantation in several hospitals and by multivariate logistic regression that acute graft‐versus‐host disease postoperative infection, PRKDC [Hazard Ratio (HR) = 4.44; 95% CI = [1.60, 13.68]; *p* = 0.006] mRNA level correlated with the renal function after transplantation. The prediction model constructed had good predictive accuracy (C‐index = 0.886). Elevated levels of donor kidney PRKDC are associated with renal dysfunction after transplantation. The prediction model of renal function status for post‐transplant recipients based on PRKDC has good predictive accuracy and clinical application.

## 1 INTRODUCTION

Renal transplantation is a beneficial therapy for many patients with renal failure as well as some chronic renal diseases. However, in the early stages of transplantation, delayed graft function (DGF), or malfunction of a transplanted kidney, is a severe complication. DGF is a severe dysfunction that can give rise to a prolonged hospital stay, a high risk of acute and chronic acute graft‐versus‐host disease (GVHD), and even graft failure.^1^ DGF occurs primarily in a lack of donors and is influenced by a variety of recipients^2^ and donor factors,^3^ as well as cold ischemic time.^4^ The incidence of DGF varies considerably between countries and may be related to ethnicity, with incidence rates ranging from 21% to 70%.^5^ The most accepted definition of DGF is at least one dialysis session in the first week after transplantation.^6^ Even though a few risk factors for DGF have been identified through recent studies, the pathogenesis of the disease is not fully understood.

The PRKDC gene is located on chromosome 8q11. The PRKDC gene encodes the DNA‐dependent protein kinase catalytic subunit (DNA‐PKcs), a member of the phosphatidylinositol 3‐kinase‐related kinase family, a serine/threonine protein phosphorylation kinase.^7^ The mutation rates of PRKDC and the expression levels of DNA‐PKcs vary significantly in various tumours. Chen et al. performed a statistical analysis of the mutation rates of PRKDC reported in The Cancer Genome Atlas and Chinese population databases; the authors found that PRKDC had high mutation rates in several tumours, including gastric, colorectal and endometrial cancers, with a high correlation with microsatellite instability‐high correlation with microsatellite instability.^8^ However, the role of PRKDC in the donor kidney and its effect after transplantation has not been reported.

With the technological advances in gene high‐throughput technology and proteomics, combined with bioinformatics approaches, access to validated biomarkers for diagnosis and patient survival has been facilitated. Bioinformatics‐based multi‐gene co‐expression analysis is critical. WGCNA is a multigene analysis method first proposed in 2008.^9^ Instead of individual genes or isolated biomarkers, WGCNA focus on gene co‐expression modules. It associates them with specific features, improving the efficacy of identifying potentially valuable bioregulatory targets.^9^ Gene differential expression analysis is an effective way to find the driver genes of a disease.^10^ In summary, the simultaneous use of differential gene expression analysis and WGCNA enables the search for potential biomarkers that affect the functional status of specific organs.

In this project, we obtained high‐throughput data from the GEO database for 192 kidney transplant donor kidneys. We used differential gene expression analysis and WGCNA to search for potential genes that affect post‐transplant kidney function. In addition, we used differential gene enrichment analysis and PPI networks to screen out central genes. We proceeded to examine the potential role of PRKDC in influencing post‐transplant renal function through comprehensive bioinformatics analysis and validated the results with 122 patients in our medical centre. We performed a logistic regression analysis based on the screened target genes to screen for high‐risk factors and constructed a predictive model for post‐transplant renal functional status based on these genes for clinical application.

## 2 METHODS

### 2.1 Data filtering

Clinical data and gene data related to kidney transplant donor kidneys were obtained from the GEO database (https://www.ncbi.nlm.nih.gov/gds). Data filtering was performed using a function ‘rpmk’ in the edgeR package.^11^ After searching the database, this study used the R function ‘GEOquery’ to download a collection of clinical data such as renal functional status after kidney transplantation (GSE147451)^12^ for a total of 192 patients.

### 2.2 WGCNA analysis

To increase the accuracy of the joint analysis of multiple genes, genes used for WGCNA were filtered. We used the R function ‘WGCNA’ to perform multiplex analysis between gene expression data profiles of GEO, grouping genes that are co‐highly expressed into different modules.^13^ ‘PickSoftThreshold’ was used to build a scale‐free network. After performing Pearson correlation analysis, we made a similarity matrix. We then correlated the previously filtered modules with their corresponding clinical features to identify the needed functional modules.

### 2.3 Differentially expressed genes (DEG) analysis

To clarify donor DEGs between different renal functional states in patients after renal transplantation, we applied the R function ‘limma’ in the database. the criteria for screening DEGs were adj. *p* < 0.05 and |logFC| ≥ 1.0. the R packages VennDiagram and ggplot2 were applied to plot Venn and Volcano plots.^14^

### 2.4 Pathway and function enrichment analysis

We enriched specific genes for specific functional parts or signalling pathways to clarify the function of GEO differential genes. Genome Encyclopedia (KEGG) and Gene Ontology (GO) pathway analysis were performed by the cluster profile R package.^15^

### 2.5 Identification of hub genes and construction of PPI and

We have constructed the PPI network through the STRING database (https://string‐db.org/). The network was analysed and established with Cytoscape software (v 3.8.1) using ≥0.4 points as the extraction value. In the network, edges represent protein interactions, and nodes represent proteins. To specify the hub genes in the network, we used the maximum cluster centrality algorithm calculated by CytoHubba's plug‐in. The top four genes were set as hub genes.

### 2.6 Analysis of the expression levels of hub genes

To further verify the accuracy of the screened genes, we analysed the expression of these critical genes in different renal functional states of patients after renal transplantation. A bar graph represented the expression of each gene in other renal functional forms. The statistical significance between the two groups was determined by Student's *t*‐test subject to normality (*p* < 0.05).

### 2.7 RT‐qPCR analysis

RT‐qPCR was performed using QuantStudio 3 platform with SYBR Green. qPCR was started at 50°C for 2 min and 95°C for 10 min, followed by 40 cycles of amplification at 95°C for 15 s and 60°C for the 60 s. Reverse transcription was performed at 25°C for 10 min, 42°C for 15 min, and 85°C for 5 min. mRNA levels were standardized with GAPDH as an internal control and determined according to the 2(^−ΔΔCt^) method. The sequences used were as follows: RPL35A, forward, 5′‐TTGAAGGTGTTTACGCCCGAG‐3′ and reverse, 5′‐TGCTTCGGAATTTGGCACGA‐3′; UBQLN4, forward, 5′‐ATTCGGGTCACCGTCAAGAC‐3′ and reverse, 5′‐GCCTTAAACCTCCGGGAGATTT‐3′; GAPDH, forward, 5′‐ GAGAAGGCTGGGGCTCATTT‐3′ and reverse, 5′‐ ATGACGAACATGGGGGCATC‐3′.

### 2.8 Patients

This study included data from 122 kidney transplant patients collected from the Ninth People's Hospital of Suzhou and the First Affiliated Hospital of Soochow University between 1 February 2018, and 31 January 2021, as external validation. We collected the data on 31 February 2021. The median age was 44 (IQR 24–52), and the project included 74 (61.0%) men. Patient data, including gender, age, body mass index, primary disease, chronic disease history, dialysis method, immunosuppressant use, aGVHD, infection, preoperative and postoperative creatinine, Ccr and PRKDC expression levels were selected for analysis. Glomerular filtration rate ≤ 50 mL/min was regarded as poor renal function group. Informed consent was obtained from all patients' immediate family members or patients' own. All research projects conformed to the guidelines of the Soochow University Ethics Committee and followed the Declaration of Helsinki (ID202214523). The subjects included in the external validation portion of this study were subjected to the same conditions as the cases in the GSE147451 data set.

### 2.9 Statistical analysis

Missing values, accounting for ≤5.0% of the data, were estimated using the ‘mice’ package^16^ in RStudio (R version 3.5.4)^17^ in RStudio, version 3.5.4. The random forest method was utilized for this estimation. Categorical variables were analysed using proportions and χ^2^ tests. Skewed variables were expressed as medians with interquartile ranges. Kruskal‐Wallis tests or one‐way anova were used for comparisons between groups. COX regression models were employed for both univariate and multivariate post‐transplant renal functional status analyses, which were adjusted accordingly. COX regression we utilized is consistent with the pH hypothesis. A multifactorial regression model was utilized to confirm risk factors and predict renal functional status after kidney transplantation. To ensure the validity of our analysis, we included significant variables from the multifactorial regression in the nomogram. The weights of each variant were confirmed and nomograms were generated and internally validated via a 1001‐bootstrap method18. The C statistic was calculated using the ‘rms’ package in R software. Statistical analyses were performed using several R packages, namely ‘PredictABEL’, ‘rms’, ‘risk regression’, ‘survminer’, and ‘ggplot2’19. A significance level of *p* < 0.05 was used throughout the analysis.^18^

### 2.10 Patient and public involvement

The study was a comprehensive study based on a public database and local hospital cases, and the project evaluated renal reconstruction and function after kidney transplantation. The public—that is a statutory health insurer, physicians, local medical managers and patient representatives—was involved in the design and implementation of the entire project. In addition, practitioners (physicians, local health care workers, health care administrators) and scientific experts were involved in the discussions of this study. The results of the study and the project as a whole will be disseminated to participants through practise‐oriented publications and newsletters.

## 3 RESULTS

**FIGURE 1:**
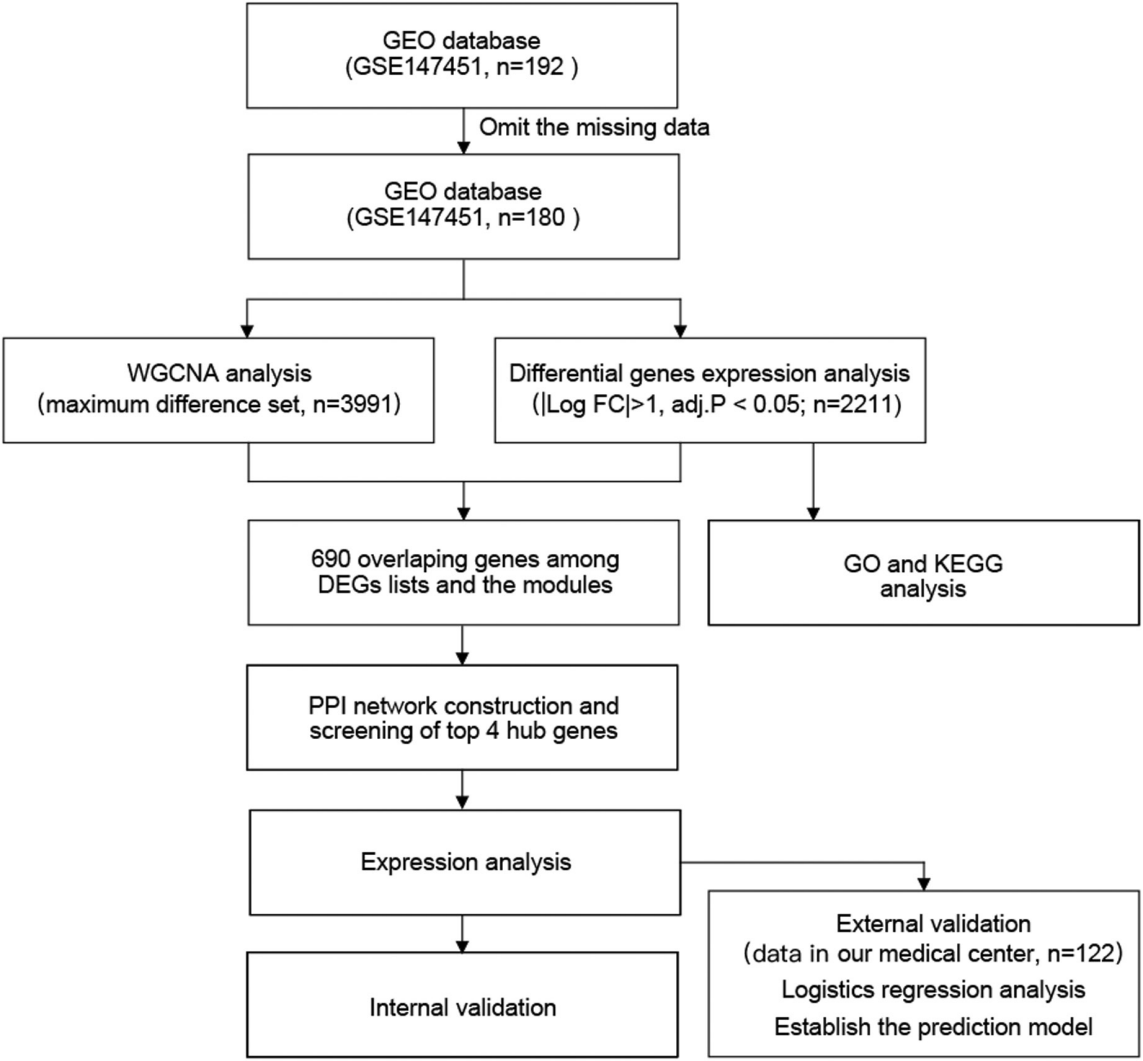
Project design and flow chart of this study.

**FIGURE 2:**
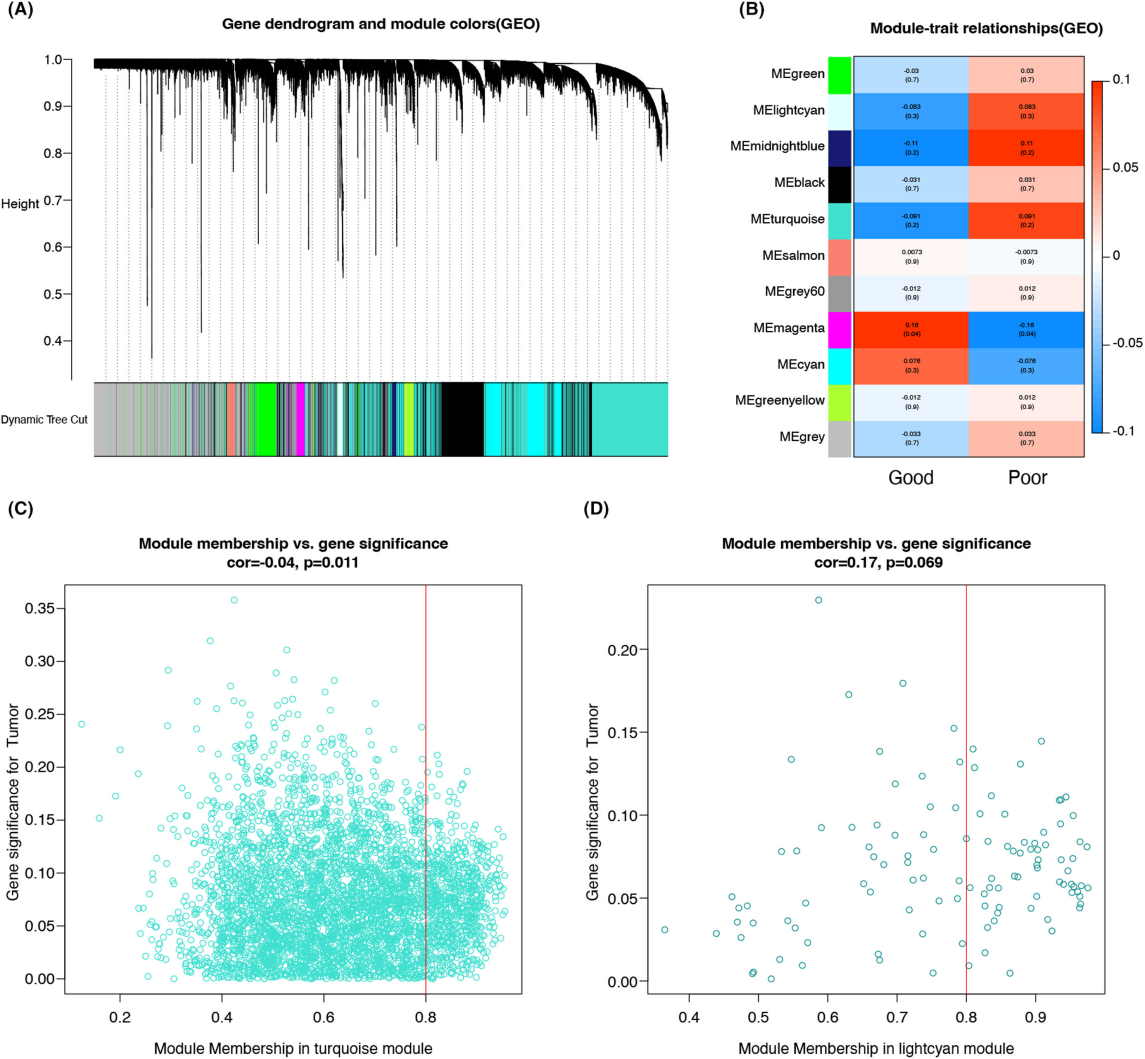
Identifying modules related to clinical information in the GEO data set (GSE147451). Cluster dendrograms of co‐expression network modules are sorted by hierarchical clustering of genes GEO data set (A). Each module was assigned a different colour. Module–trait relationships in the GEO data set (B). Each row corresponds to a coloured module, and each column corresponds to a clinical trait (normal and abnormal renal function after transplantation): Midnight blue module (C) and the turquoise module (D) corresponding correlations and *p*‐values.

The flow chart for this project is shown in Figure 1. We performed a WGCNA analysis from the GEO database (GSE147451) to identify the critical genomes of donor kidneys that affect renal function after kidney transplantation. Each colour represents a module. Here, 13 modules in the data set (Figure 2A) were confirmed. The results of the analysis do not indicate any significant outliers. Figure 2B shows module–trait relationships, indicating that the turquoise module (Figure 2C) and the lightcyan module (Figure 2D) were highly discriminatory between different post‐transplant renal functional states (turquoise module: cor = 0.04, *p* = 0.011, lightcyan module: cor = 0.17, *p* = 0.069). Other modules are displayed in the supplemental materials.

**FIGURE 3:**
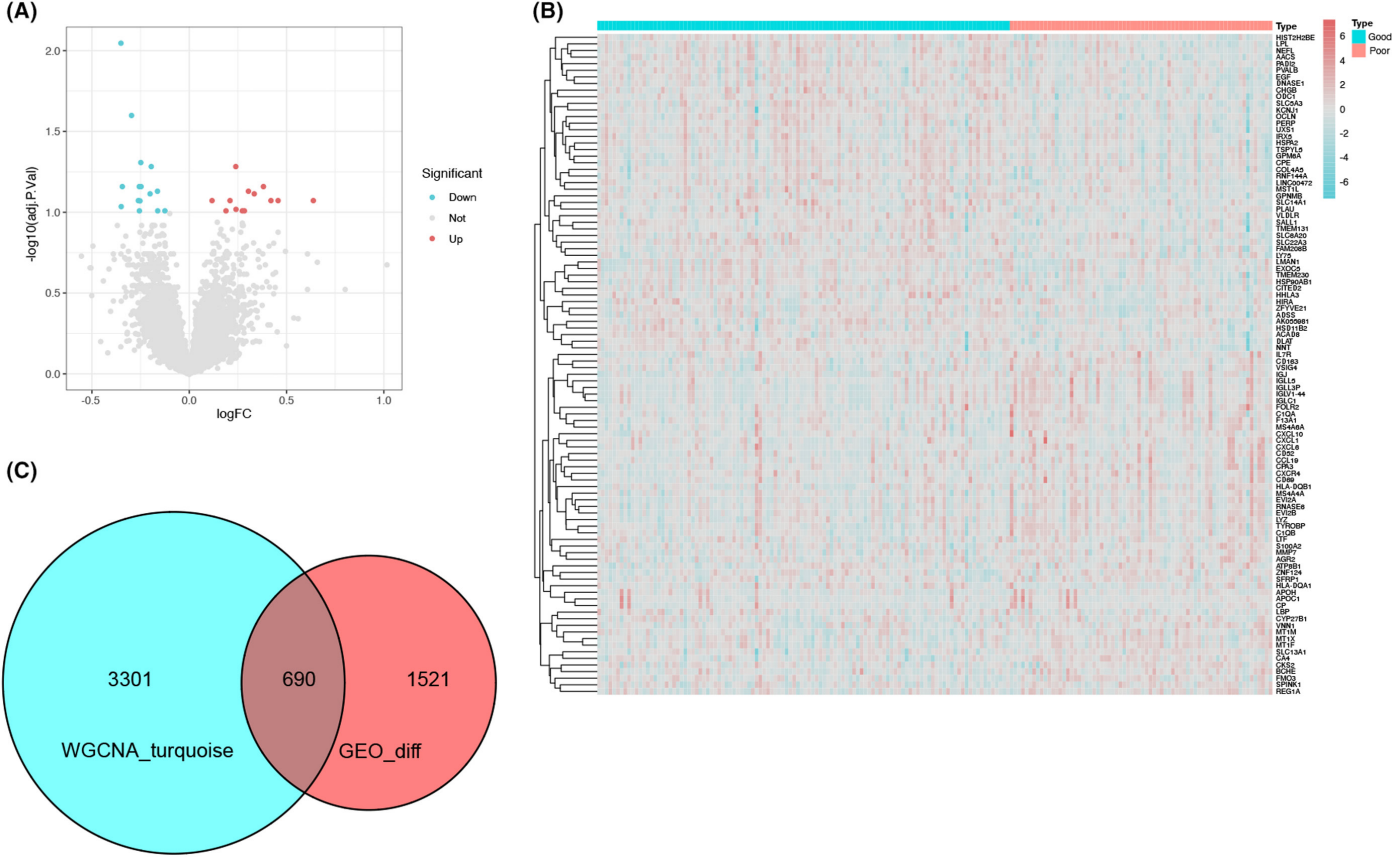
DEGs were identified in the kidney transplant donor data set of GSE147451 with logFC| ≥ 1.0 and adj. *p* < 0.05 as the cut‐off criteria. (A) Volcano map of DEGs in the GEO data set. (B) Heat map of the top 50 DEGs in the GSE147451 data set. (C) Venn diagram of genes between the DEG list and the co‐expression module.

We have found that 2211 DEGs in the GEO data set (Figure 3A) were selected via the ‘limma’ package, using adj. *p* < 0.05 and |logFC| ≥ 1.0 as the cut‐off criteria. We decided on the top 50 genes based on expression differences and statistical significance to plot the heat map (Figure 3B). Sixty‐six genes were obtained from these modules according to WGCNA. Finally, 690 genes were obtained at the intersection of the DEGs and midnight‐blue modules involving genes obtained from the GEO database (Figure 3C).

**FIGURE 4:**
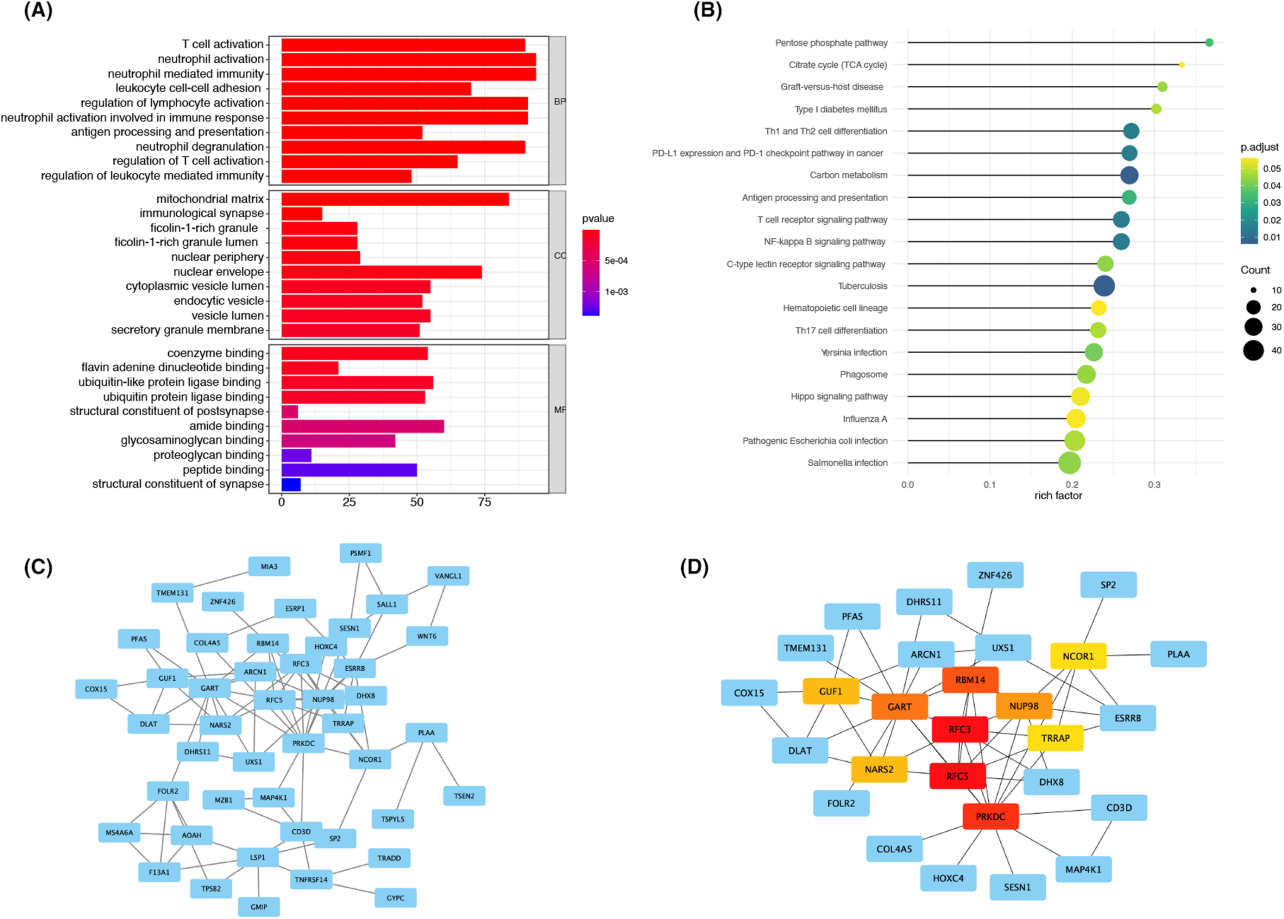
GO (A) and KEGG (B) enrichment analysis of genes in the GEO database. The colours represent adjusted *p* values, and the size of the spots represents the number of genes. Visualization of PPI networks and candidate hub genes. (C) PPI network of genes between the DEG list and the two co‐expression modules. Blue nodes represent genes. Edges indicate the interaction relationships between the nodes. (D) Identification of central genes in the PPI network using the MCC algorithm.

To fully understand the functions of these genes in the co‐expression module, GO (Figure 4A) and KEGG (Figure 4B) analyses were performed on the differential genes in GEO using the R package ‘ClusterProfiler’. GO investigation classified genes into three categories: cellular components (CC), molecular functions (MF), and biological processes (BP). The genes involved in T‐cell activation and neutrophil activation were enriched in BP. The CC category involved several genes in the mitochondrial matrix and immunological synapse. In addition, the amide binding and ubiquitin‐like protein ligase binding were mainly engaged in MF. KEGG displayed that genes in Graft versus host disease were enriched.

**FIGURE 5:**
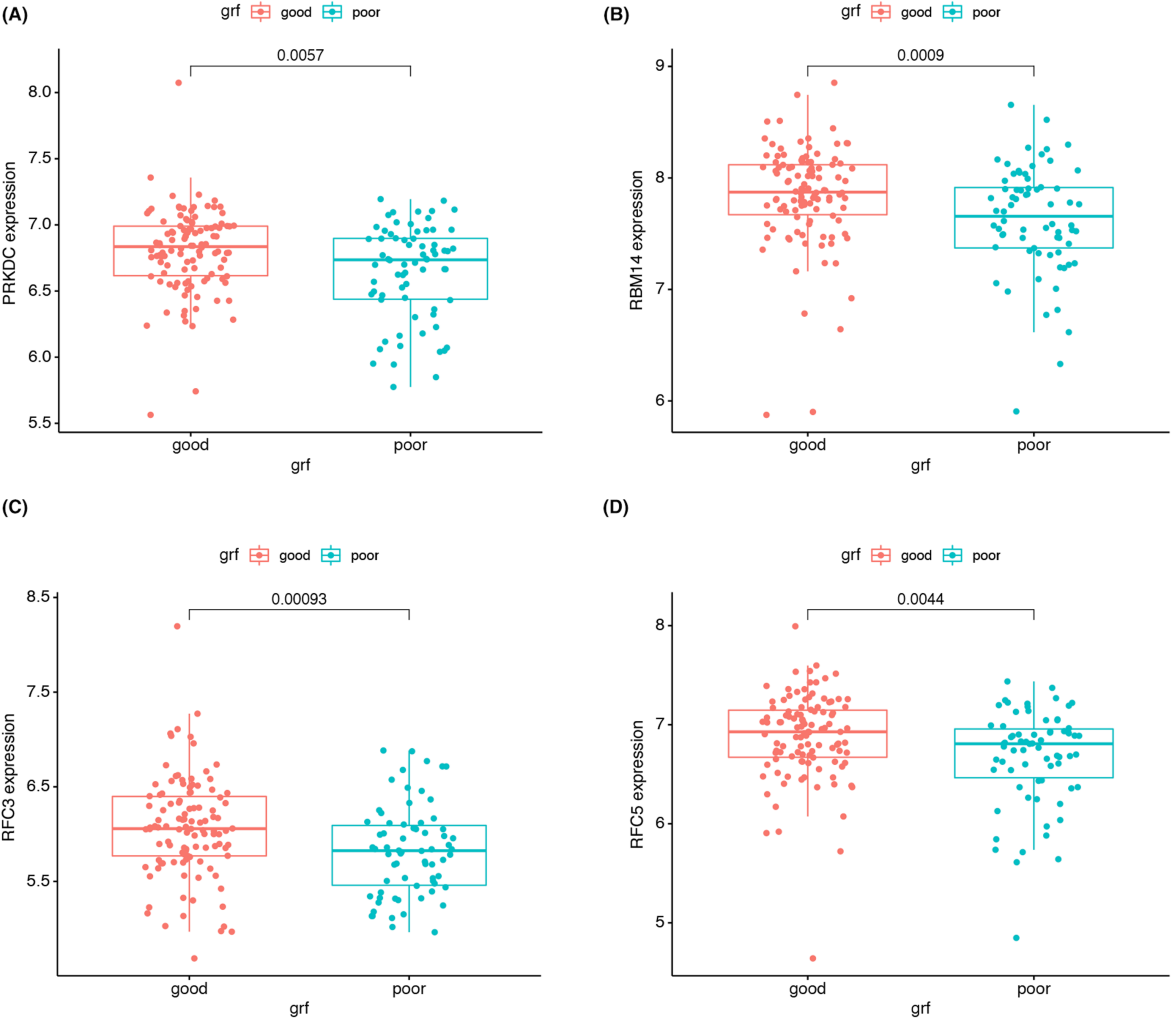
Histogram of the differential expression of PRKDC (A), RBM14 (B), RFC3 (C) and RFC5 (D) in different renal functional states after renal transplantation.

We analysed the STRING database PPI to investigate the relationship between co‐expression modules and DEGs. We plotted a PPI network with 320 edges and 219 nodes in Figure 4C. In addition, the top four hub genes with MCC scores in the PPI were calculated using the CytoHubba plugin. These genes are PRKDC, RFC5, RFC3 and RBM14 (Figure 4D). All four essential genes were highly expressed in patients with poor post‐transplant renal function (Figure 5).

To further clarify the effect of the above genes on renal function after kidney transplantation, we collected clinical data from 122 kidney transplant patients at the medical centre. We examined the expression levels of the above genes. The clinical baseline data for these patients are shown in Table 1. Specifically, 122 patients who underwent kidney transplantation between 1 February 2018, and 31 January 2021, to the follow‐up endpoint (1 December 2021) were included in this study. Among them, the poorer renal function was 30 (GFR < 50 min/L). Among these patients, the median BMI was 21.52. 92 (75%) patients had the hypertensive disease. 66 (54%) patients received preoperative haemodialysis, while 48 (39%) patients received peritoneal dialysis. The overall mean level of PRKDC was 13.98 ± 7.88. aGVHD occurred in 39 (32%) patients and infection occurred in 58 (48%) patients.

**TABLE 1:** Study participant characteristics at enrolment.

Variables	Total (*n* = 122)	Glomerular filtration rate		
		>50 mL/min (*n* = 92)	≤50 mL/min (*n* = 30)	*p*‐value
Age, median (Q1, Q3)	44 (34, 52)	40.5 (34, 51)	47 (43.25, 52.75)	0.027*
Gender, *n* (*%*)				0.465
Male	74 (61)	58 (63)	16 (53)	
Female	48 (39)	34 (37)	14 (47)	
Chronic disease, *n* (%)				0.321
None	18 (15)	13 (14)	5 (17)	
Hypertension	92 (75)	72 (78)	20 (67)	
Diabetes	6 (5)	4 (4)	2 (7)	
Others	6 (5)	3 (3)	3 (10)	
Primary disease, *n* (%)				0.644
Chronic renal failure	78 (64)	57 (62)	21 (70)	
Chronic glomerulonephritis	21 (17)	16 (17)	5 (17)	
Others	23 (19)	19 (21)	4 (13)	
Dialysis method, *n* (%)				0.944
None	8 (7)	6 (7)	2 (7)	
Haemodialysis	66 (54)	49 (53)	17 (57)	
Peritoneal dialysis	48 (39)	37 (40)	11 (37)	
BMI, median (Q1, Q3)	21.52 (19.41, 24.03)	21.4 (19.46, 24.07)	21.76 (19.44, 23.79)	0.912
FK506 use, *n* (%)				0.22
No	25 (20)	16 (17)	9 (30)	
Yes	97 (80)	76 (83)	21 (70)	
MMF use, *n* (%)				0.523
No	57 (47)	45 (49)	12 (40)	
Yes	65 (53)	47 (51)	18 (60)	
CSA use, *n* (%)				0.106
No	108 (89)	84 (91)	24 (80)	
Yes	14 (11)	8 (9)	6 (20)	
ATG use, *n* (%)				1
No	120 (98)	90 (98)	30 (100)	
Yes	2 (2)	2 (2)	0 (0)	
Cr before surgery, median (Q1, Q3) (μmol/L)	934.05 (790.95, 1180.45)	976 (796.42, 1183.92)	842 (747.65, 1061.2)	0.248
aGVHD, *n* (%)				<0.001***
No	83 (68)	72 (78)	11 (37)	
Yes	39 (32)	20 (22)	19 (63)	
Infection, *n* (%)				0.002**
No	64 (52)	56 (61)	8 (27)	
Yes	58 (48)	36 (39)	22 (73)	
Cr after surgery, median (Q1, Q3) (μmol/L)	99.85 (87.25, 126.95)	96.05 (77.3, 112.92)	141 (108.4, 189.52)	<0.001***
Ccr after surgery, median (Q1, Q3) (mL/min)	63.15 (50.13, 80.43)	69.61 (61.5, 87.16)	40.3 (25.43, 45.2)	<0.001***
PRKDC expression, mean ± SD	13.98 ± 7.88	11.7 ± 6.13	20.98 ± 8.59	<0.001***

Abbreviations: aGVHD, acute graft‐versus‐host disease; ATG, antithymocyte globulin; BMI, body mass index; Ccr, creatinine clearance rate; Cr, creatinine; CSA, Ciclosporin A; MMF, mycophenolate mofetil.

* *p* < 0.05

** *p* < 0.01

*** *p* < 0.001.

**FIGURE 6:**
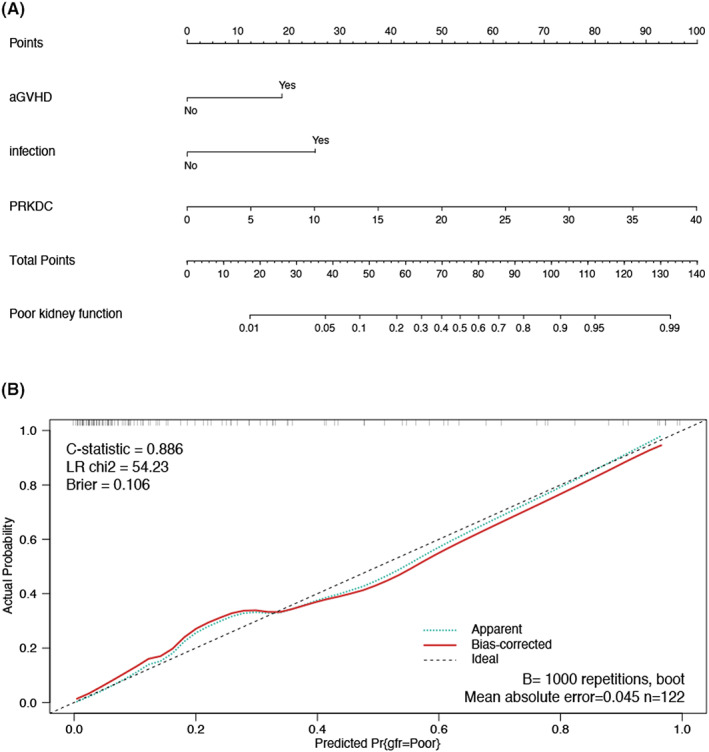
Nomogram predicting the risk of bleeding renal insufficiency in patients after renal transplantation. (A) Nomogram for predicting renal insufficiency in patients. (B) Validity of calibration curves in estimating patient prognosis.

Subsequently, by univariate logistics regression, we included variables (*p* < 0.2) in the multifactorial analysis. It showed that aGVHD [HR = 5.66; 95% CI = [2.05, 16.73]; *p* < 0.001], infection [HR = 5.10; 95% CI = [1.85, 15.67]; *p* = 0.003] and PRKDC [HR = 4.44; 95% CI = [1.60, 13.68]; *p* < 0.006] were all independent risk factors for post‐transplant renal insufficiency (Table 2). Then, independent risk factors screened by the multifactorial analysis were selected to construct a predictive model of renal functional status after kidney transplantation using nomogram (Figure 6A). Nomogram predicted the risk of renal dysfunction with a C‐statistic of 0.886. A fitted calibration curve is in the internal validation in Figure 6B.

**TABLE 2:** Cox regression analysis of hazard ratio on renal function after renal transplantation.

Variables	Univariate analysis		Multivariate analysis	
	Hazard ratio (95% CI)	*p*‐value	Hazard ratio (95% CI)	*p*‐value
Gender, female versus male	1.49 [0.64, 3.44]	0.346	‐	‐
FK506 use, yes versus no	0.49 [0.19, 1.30]	0.142	1.14 [0.20, 8.03]	0.885
MMF use, yes versus no	1.44 [0.63, 3.38]	0.397	‐	‐
CSA use, yes versus no	2.62 [0.80, 8.30]	0.1	3.26 [0.38, 33.81]	0.295
ATG use, yes versus no	‐	0.989	‐	‐
aGVHD, yes versus no	6.22 [2.59, 15.64]	<0.001***	5.66 [2.05, 16.73]	0.001**
Infection, yes versus no	4.28 [1.78, 11.20]	0.002**	5.10 [1.85, 15.67]	0.003**
Age, >60 versus ≤60 (year)	0.76 [0.04, 5.39]	0.808	‐	‐
Chronic disease, hypertension versus none	0.56 [0.23, 1.41]	0.204	‐	‐
Primary disease, chronic renal failure versus others	1.43 [0.60, 3.62]	0.427	‐	‐
Dialysis method, haemodialysis versus others	1.15 [0.50, 2.67]	0.745	‐	‐
BMI, >24 versus ≤24	0.97 [0.37, 2.41]	0.957	‐	‐
Cr before surgery, >934 versus ≤934 (μmol/L)	0.49 [0.20, 1.12]	0.096	0.58 [0.20, 1.60]	0.293
PRKDC expression, >12.81 versus ≤12.81	3.74 [1.56, 9.76]	0.004**	4.44 [1.60, 13.68]	0.006**

Abbreviations: aGVHD, acute graft‐versus‐host disease; ATG, antithymocyte globulin; BMI, body mass index; Ccr, creatinine clearance rate; Cr, creatinine; CSA, Ciclosporin A; MMF, mycophenolate mofetil.

** *p* < 0.01

*** *p* < 0.001.

## 4 DISCUSSION

Renal transplantation is the therapy of choice for patients with renal failure. However, DGF is a severe post‐transplant complication. It can give rise to increased risk of acute and chronic rejection, prolonged hospitalization and graft failure.^19^

In this study, we analysed 192 patients in the GEO data set. Based on a comprehensive bioinformatics analysis, we obtained 690 lists of DEGs, and modules crossed by overlapping genes from the GEO data set (GSE147451). GO and KEGG analysis were performed using the function “ClusterProfiler” of R software. Immediately after, the four genes with the highest MCC scores in PPI were screened, including PRKDC, RFC5, RFC3 and RBM14. These essential genes impact the renal functional status of patients after kidney transplantation. PRKDC, whose high expression was associated with post‐transplant renal insufficiency. In the validation phase, we collected clinical data and gene expression assays from 122 kidney transplant patients in our hospital. Four independent risk factors, aGVHD, infection and PRKDC, were screened by multifactorial analysis, and a prediction model was established focusing on these genes, which showed good prediction accuracy.

Recently, gene networks have been increasingly used for bioinformatics analysis. WGCNA is a efficacy method to analyse the expected effects of multiple genes among revealed genomic data.^20^ WGCNA has been applied in various biological studies to confirm therapeutic targets or candidate biomarkers, especially in tumours.^21^, ^22^, ^23^ However, the analysis of WGCNA in transplantation‐related, especially renal functional status after kidney transplantation, is currently less studied.

In the project, we focused on the hub gene PRKDC and combined it with the external validation of 122 patients in our medical centre. Both PRKDC and DNA‐PKcs affect tumorigenesis, progression, invasion and metastasis through different pathophysiological processes and further prognostic analyses have shown that DNA‐PKcs expression can help predict patient prognosis.Lu et al.^24^ after studying the effect of DNA‐PKcs expression levels on clinical staging and lymphatic and distant metastasis of colorectal cancer, further demonstrated that DNA‐PKcs expression levels were negatively correlated with the 5‐year survival rate of patients. Xing et al.^25^ analysed the gene expression profile of NSCLC cells and showed that patients with high DNA‐PKcs or ATM expression levels in the tumour sample/normal tissue sample (T/N) ratio had a significantly increased risk of death. In other cancers such as gastric, hepatocellular, breast and nasopharyngeal cancers, the expression level of DNA‐PKcs was also negatively correlated with prognosis.^26^, ^27^, ^28^, ^29^ However, there are no reports on the functional aspects of the post‐transplant kidney and our study expands on this part. And we may predict the prognosis of kidney transplantation recipients on PRKDC.

The present study has its limitations. The effect of PRKDC should be verified in vitro experiments. Then, studies on the mechanisms affecting renal insufficiency in recipients after renal transplantation should also be continued. Prediction maps also need to be confirmed using larger sample sizes. Moreover, a prospective investigation should be initiated before the clinical application of the prediction model. We are preparing to conduct further prospective clinical trials in our follow‐up studies to validate the findings of this study.

In conclusion, we identified four essential genes affecting post‐transplant renal function through comprehensive bioinformatics analysis. By differential gene expression analysis and WGCNA, PRKDC was closely associated with post‐transplant renal function. External validation demonstrated that PRKDC expression levels are an independent risk factor for predicting renal functional status in patients after renal transplantation. Furthermore, nomograms based on aGVHD, infection and PRKDC proved more effective in assessing kidney function after transplantation.

## STRENGTHS AND LIMITATIONS OF THIS STUDY

This study found that infection and aGVHD are related to renal dysfunction after transplantation. Elevated levels of donor kidney PRKDC are associated with renal dysfunction after transplantation. The prediction model of renal function status for post‐transplant recipients based on PRKDC has good predictive accuracy and clinical application. The present study has its limitations. The effect of PRKDC should be verified in vitro experiments. Then, studies on the mechanisms affecting renal insufficiency in recipients after renal transplantation should also be continued. Prediction maps also need to be confirmed using larger sample sizes. Moreover, a prospective investigation should be initiated before the clinical application of the prediction model.

## AUTHOR CONTRIBUTIONS

**Zhijun Cao:** Formal analysis (equal); investigation (equal); writing – original draft (equal); writing – review and editing (equal). **Hao Jiang:** Data curation (equal); investigation (equal); methodology (equal); visualization (equal). **Chunchun Zhao:** Resources (equal); software (equal). **Huifeng Zhou:** Data curation (equal); formal analysis (equal). **Zheng Ma:** Visualization (equal); writing – original draft (equal). **Chen Xu:** Formal analysis (equal); investigation (equal). **Jianglei Zhang:** Project administration (equal); supervision (equal). **Minjun Jiang:** Conceptualization (lead); data curation (lead); project administration (lead); writing – original draft (equal); writing – review and editing (equal). **Zhenfan Wang:** Data curation (equal); formal analysis (equal); methodology (equal).

## FUNDING INFORMATION

This work was supported by the Natural Science Foundation of Jiangsu Province (Grants Number BK20210094), the Suzhou Gusu Medical Youth Talent (GSWS2021012), Suzhou Youth Science and Technology Program KJXW2022076 and the Jiangsu Innovative and Entrepreneurial Talent Programme(JSSCBS20211560).

## CONFLICT OF INTEREST STATEMENT

The authors declare no competing financial interests.

## Supporting information


**Appendix S1:** Supporting Information

## Data Availability

Data may be obtained from a third party and are not publicly available. The data are, in large part, owned by Soochow University. The GEO data could be download in the website as follows https://www.ncbi.nlm.nih.gov/gds.
