# Supplementary material for: Up‐regulation of PRKDC was associated with poor renal dysfunction after renal transplantation: A multi‐centre analysis
Source: J Cell Mol Med. 2023 Apr 1;27(10):1362–72. doi: 10.1111/jcmm.17737 (PMC10183702; doi:10.1111/jcmm.17737)
Supplement: Supplementary file 1 — Appendix S1: Supporting Information [file JCMM-27-1362-s001.pdf]

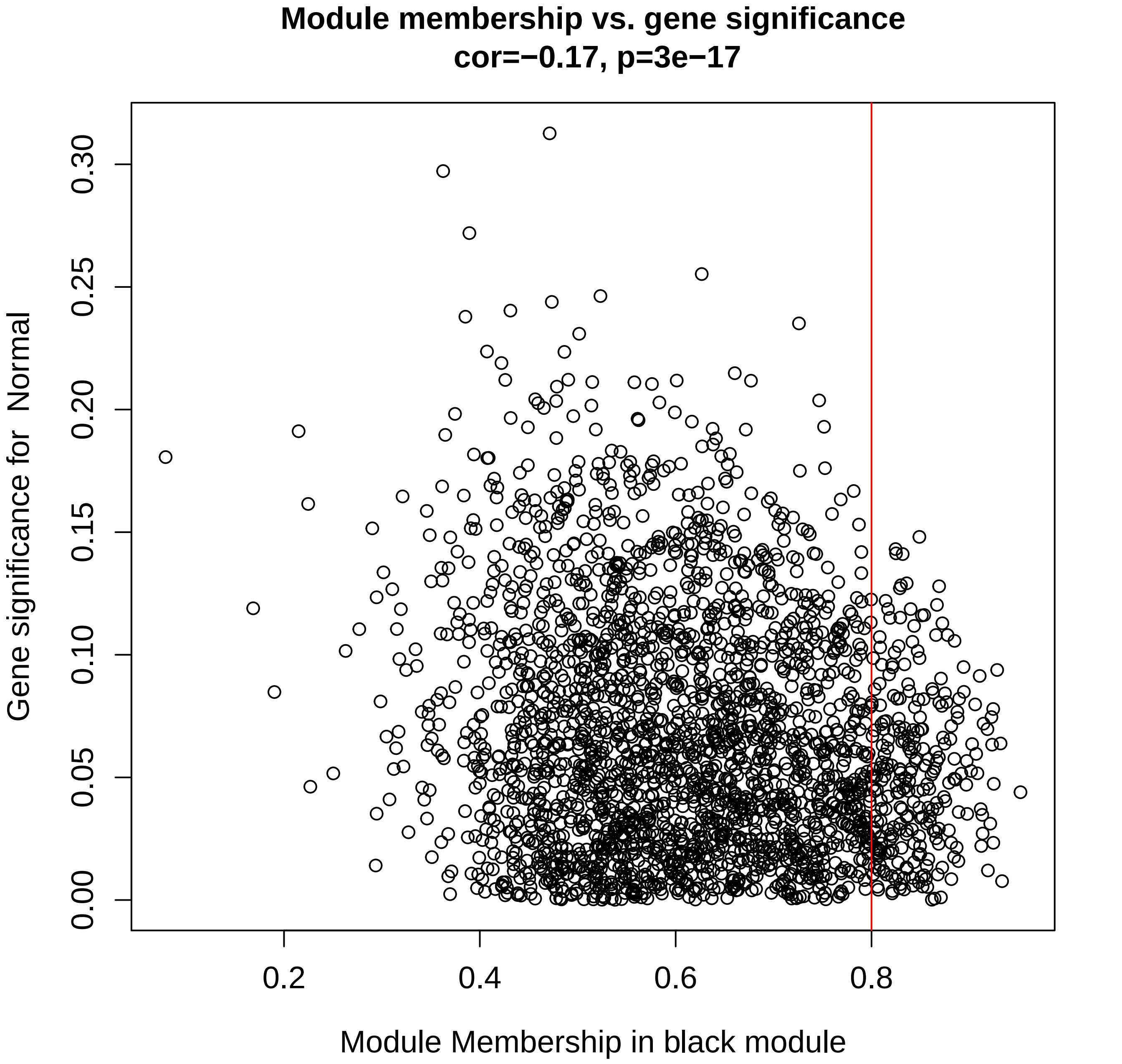

Module membership vs. gene significance
cor=−0.17, p=3e−17
Gene significance for Normal
0.00
0.05
0.10
0.15
0.20
0.25
0.30
0.2
0.4
0.6
0.8
Module Membership in black module

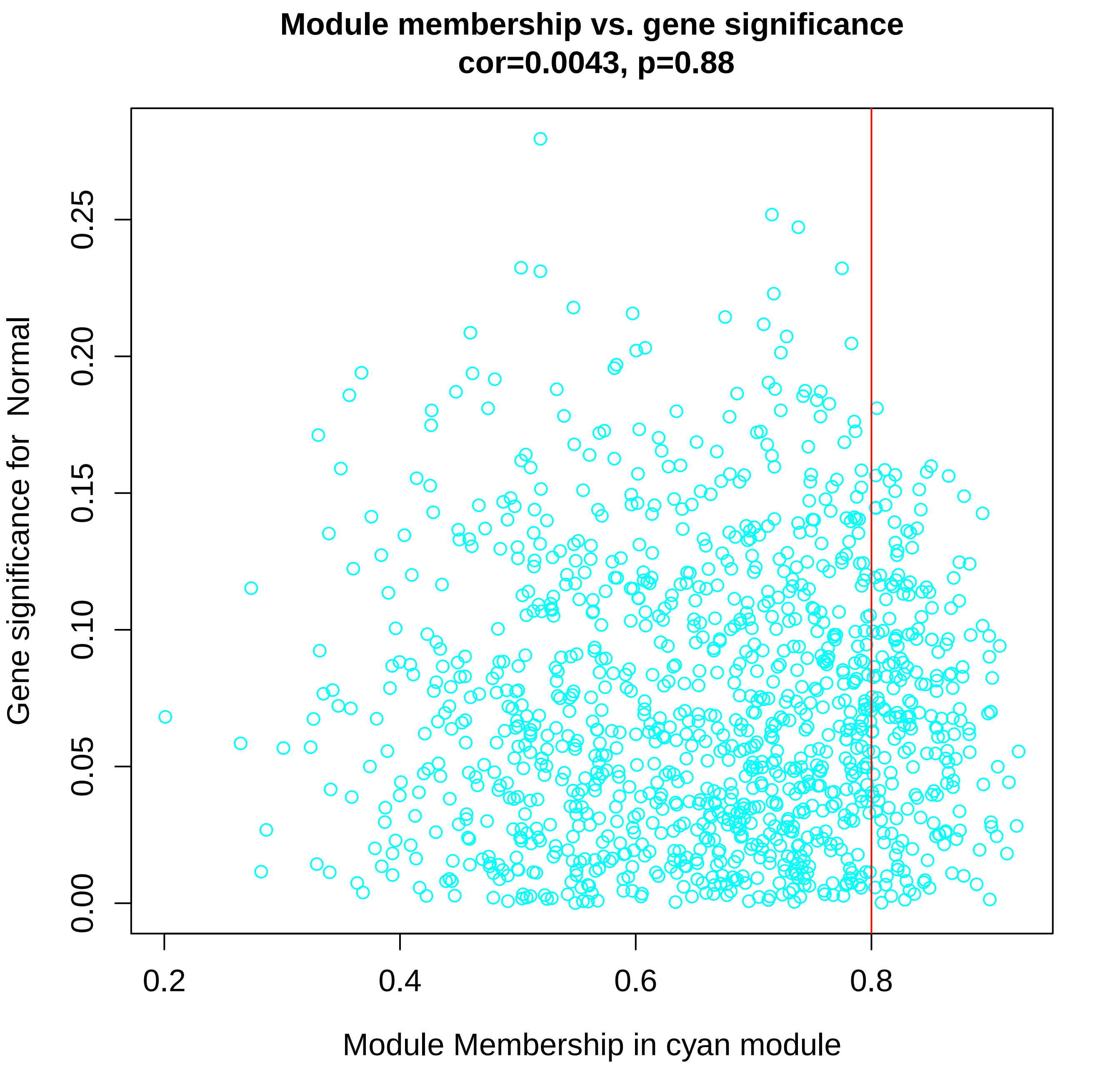
Module membership vs. gene significance
cor=0.0043, p=0.88
Gene significance for Normal
0.00
0.05
0.10
0.15
0.20
0.25
0.2
0.4
0.6
0.8
Module Membership in cyan module

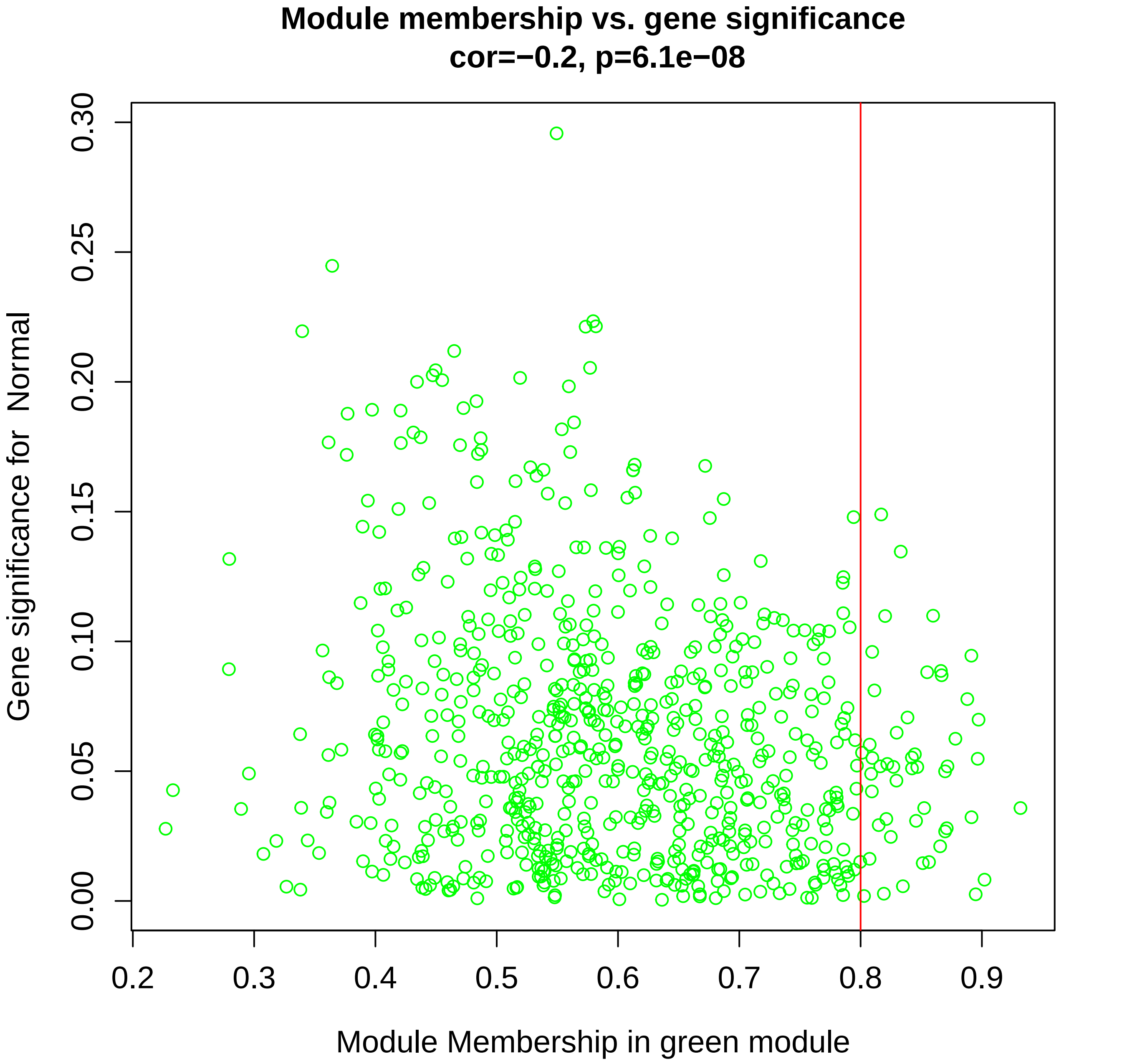

Module membership vs. gene significance
cor=−0.2, p=6.1e−08
Gene significance for Normal
0.00
0.05
0.10
0.15
0.20
0.25
0.30
0.2
0.3
0.4
0.5
0.6
0.7
0.8
0.9
Module Membership in green module

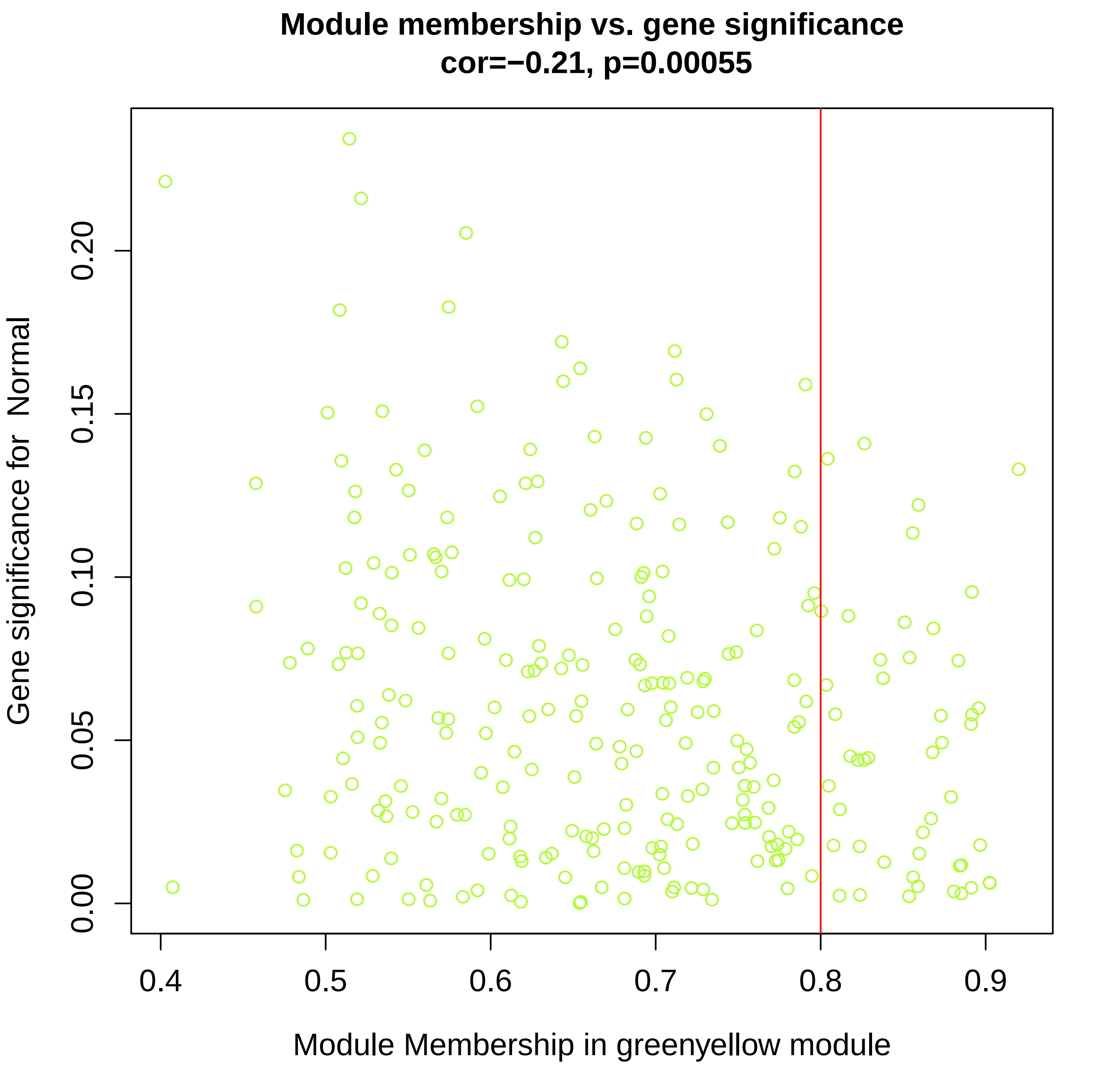

Module membership vs. gene significance
cor=−0.21, p=0.00055
Gene significance for Normal
0.00
0.05
0.10
0.15
0.20
Module Membership in greenyellow module
0.4
0.5
0.6
0.7
0.8
0.9

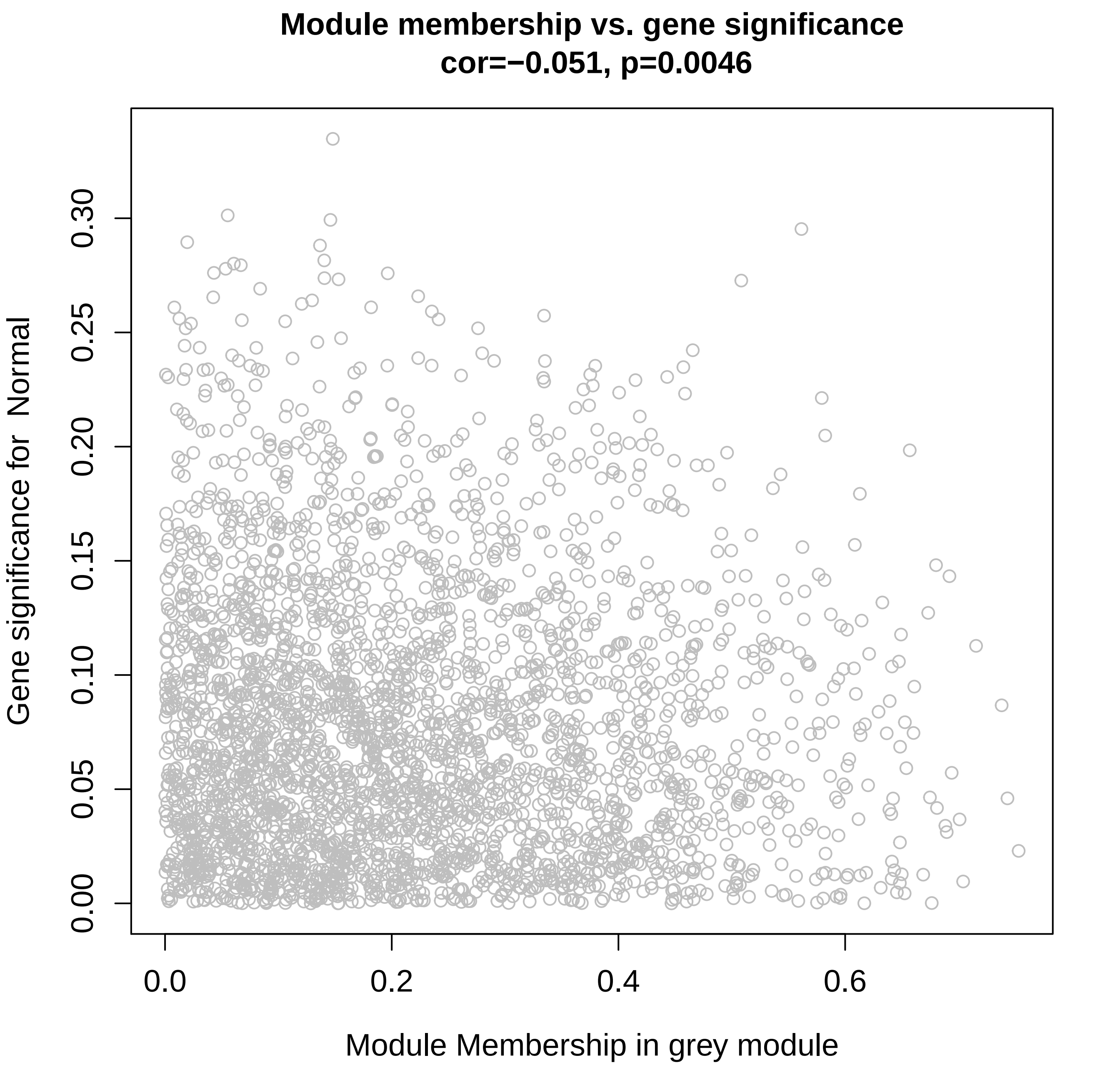
Module membership vs. gene significance
cor=−0.051, p=0.0046
Gene significance for Normal
0.00
0.05
0.10
0.15
0.20
0.25
0.30
0.0
0.2
0.4
0.6
Module Membership in grey module

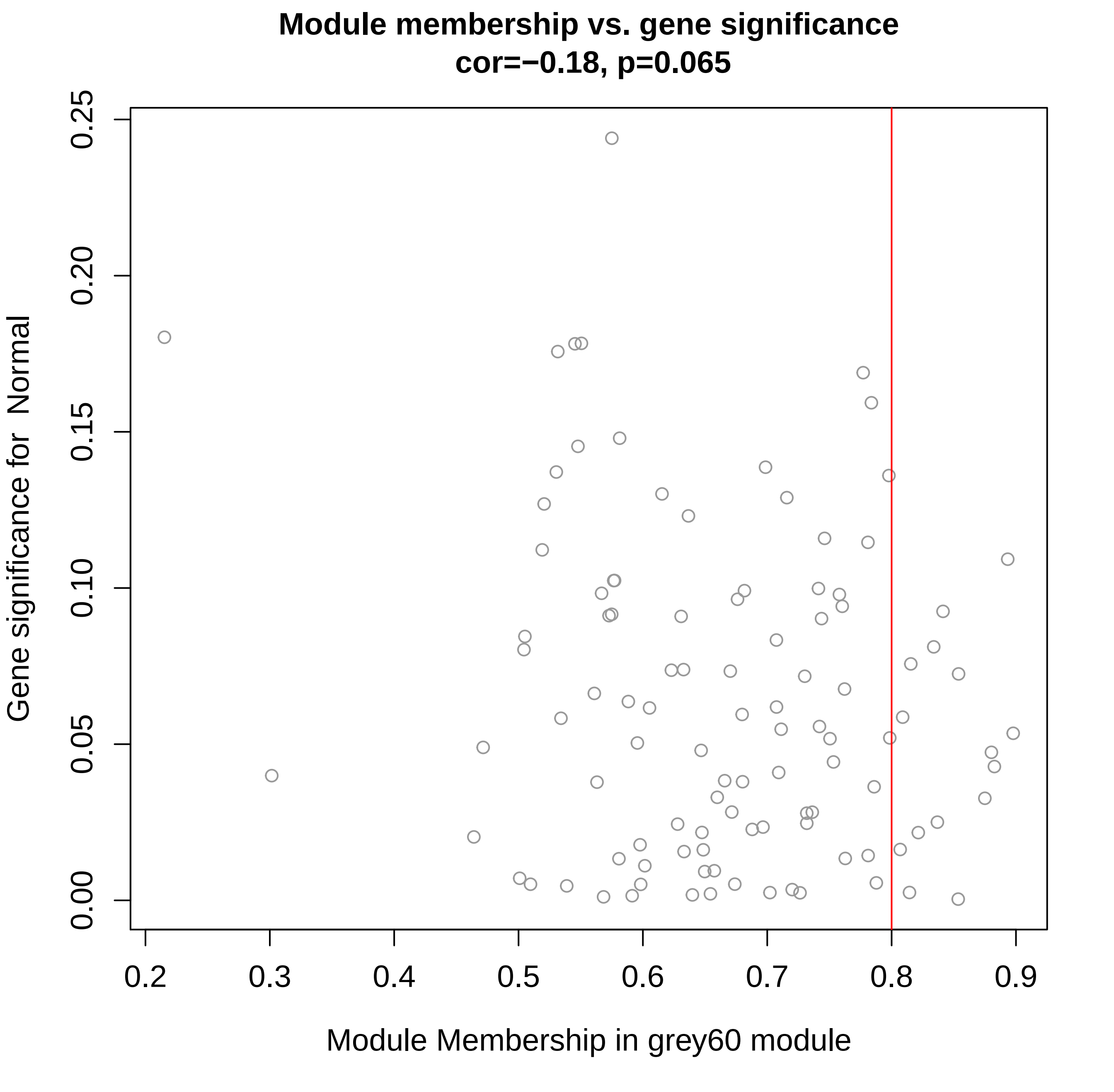

Module membership vs. gene significance
cor=−0.18, p=0.065
Gene significance for Normal
0.00
0.05
0.10
0.15
0.20
0.25
0.2
0.3
0.4
0.5
0.6
0.7
0.8
0.9
Module Membership in grey60 module

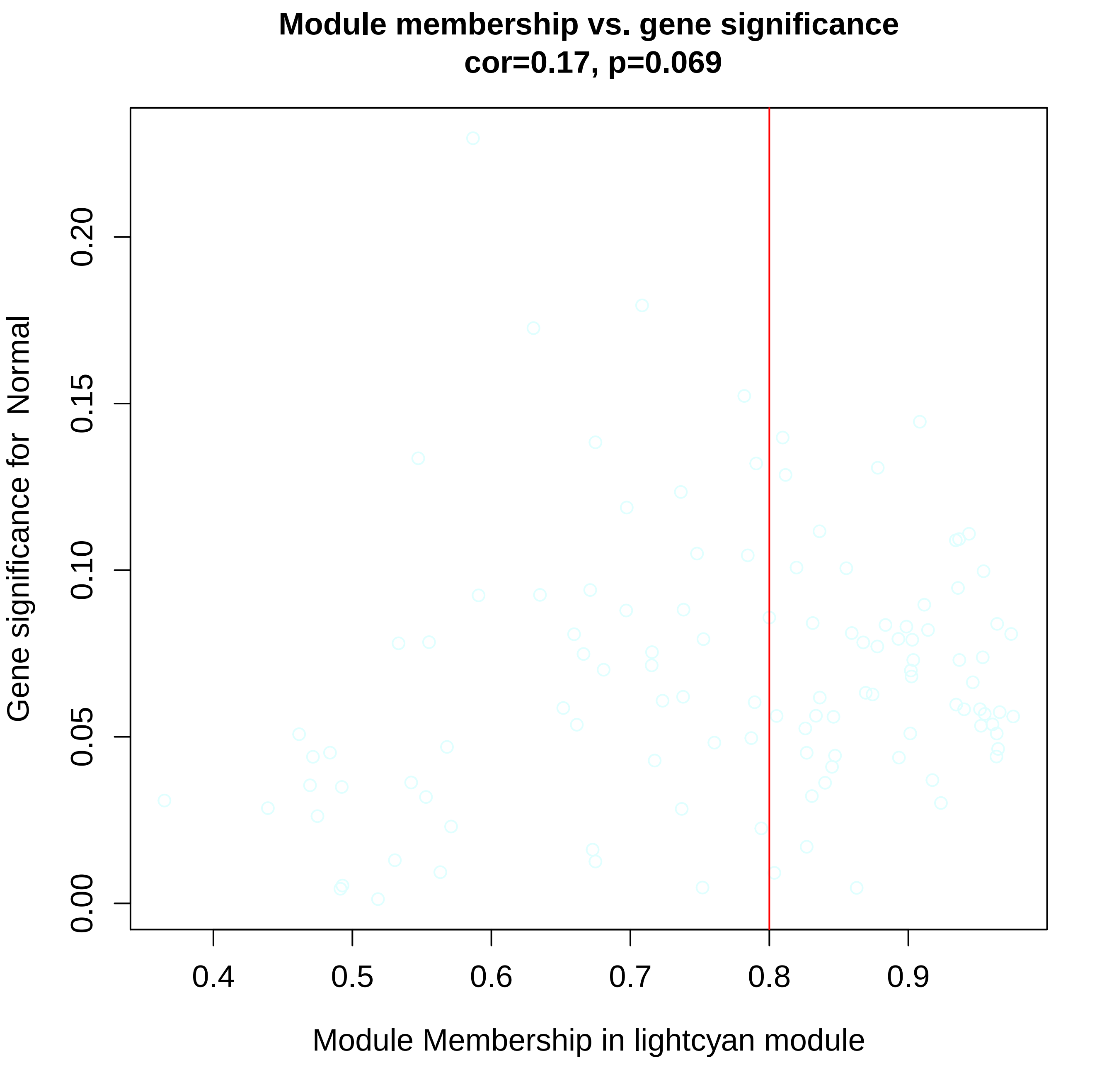
Module membership vs. gene significance
cor=0.17, p=0.069
Gene significance for Normal
0.00
0.05
0.10
0.15
0.20
0.4
0.5
0.6
0.7
0.8
0.9
Module Membership in lightcyan module

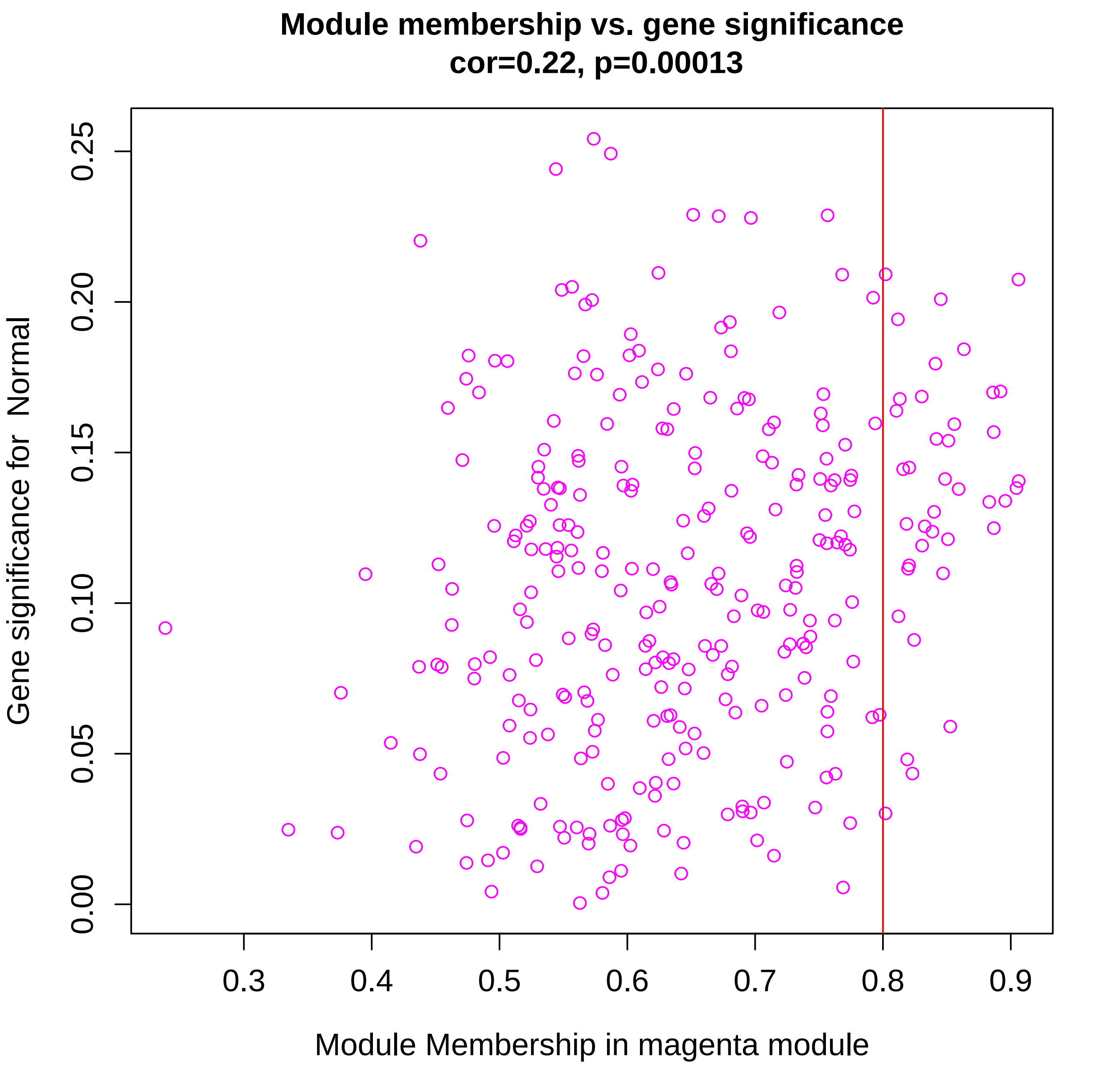
Module membership vs. gene significance
cor=0.22, p=0.00013
Gene significance for Normal
Module Membership in magenta module
0.00
0.05
0.10
0.15
0.20
0.25
0.3
0.4
0.5
0.6
0.7
0.8
0.9

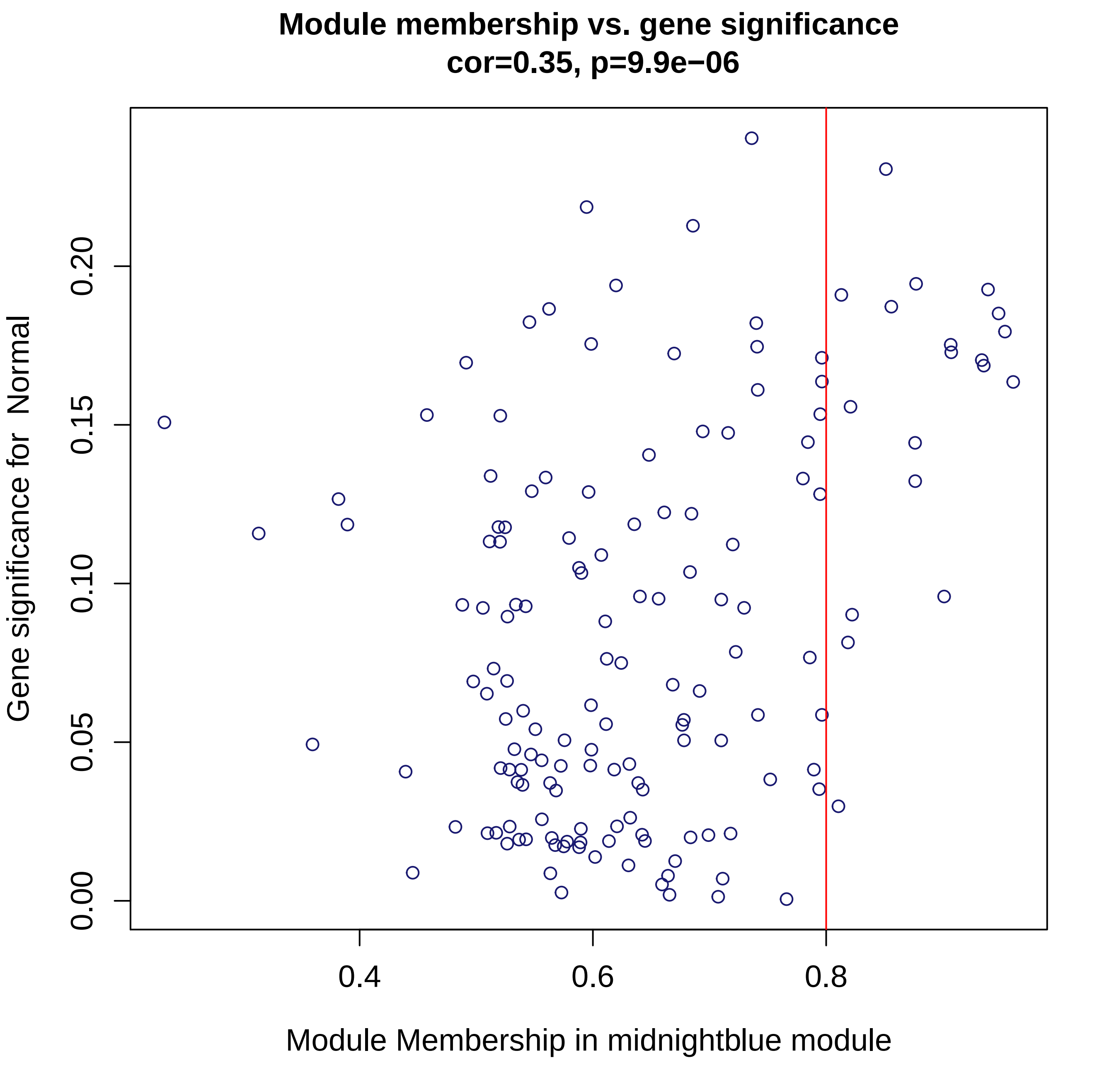
Module membership vs. gene significance
cor=0.35, p=9.9e−06
Gene significance for Normal
0.00
0.05
0.10
0.15
0.20
0.4
0.6
0.8
Module Membership in midnightblue module

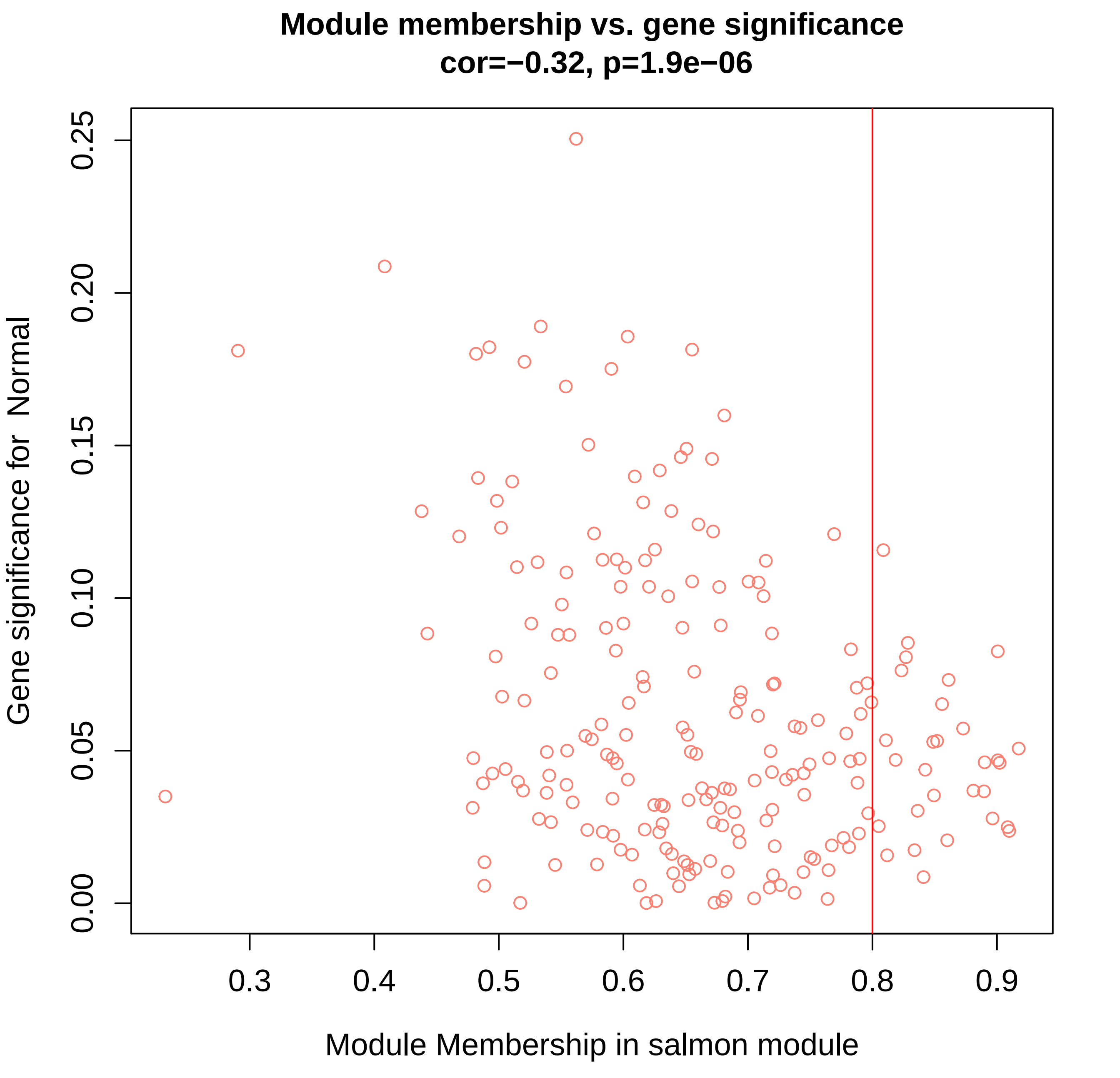
Module membership vs. gene significance
cor=−0.32, p=1.9e−06
Gene significance for Normal
Module Membership in salmon module
0.00
0.05
0.10
0.15
0.20
0.25
0.3
0.4
0.5
0.6
0.7
0.8
0.9

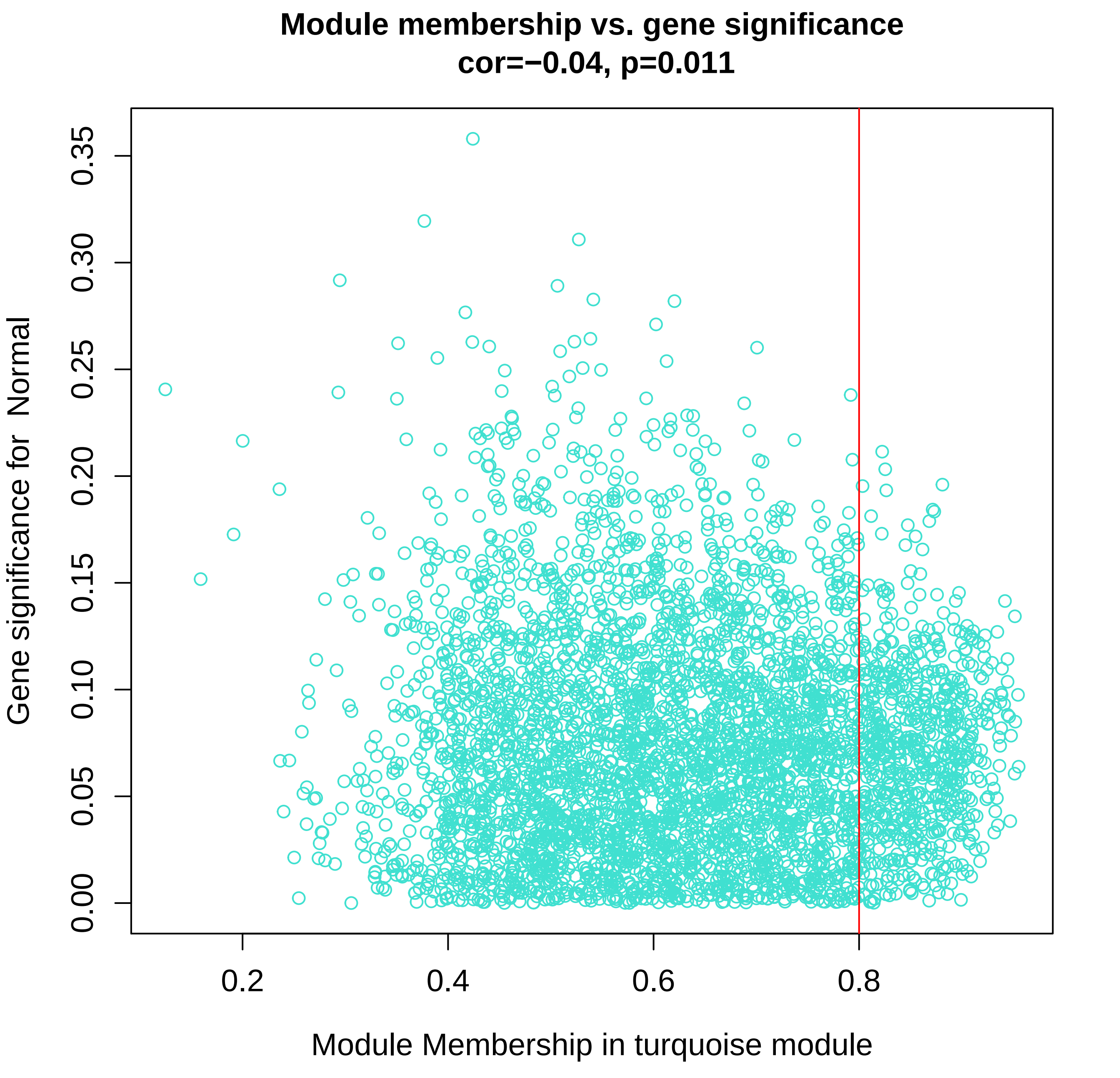

Module membership vs. gene significance
cor=−0.04, p=0.011
Gene significance for Normal
Module Membership in turquoise module
0.00
0.05
0.10
0.15
0.20
0.25
0.30
0.35
0.2
0.4
0.6
0.8

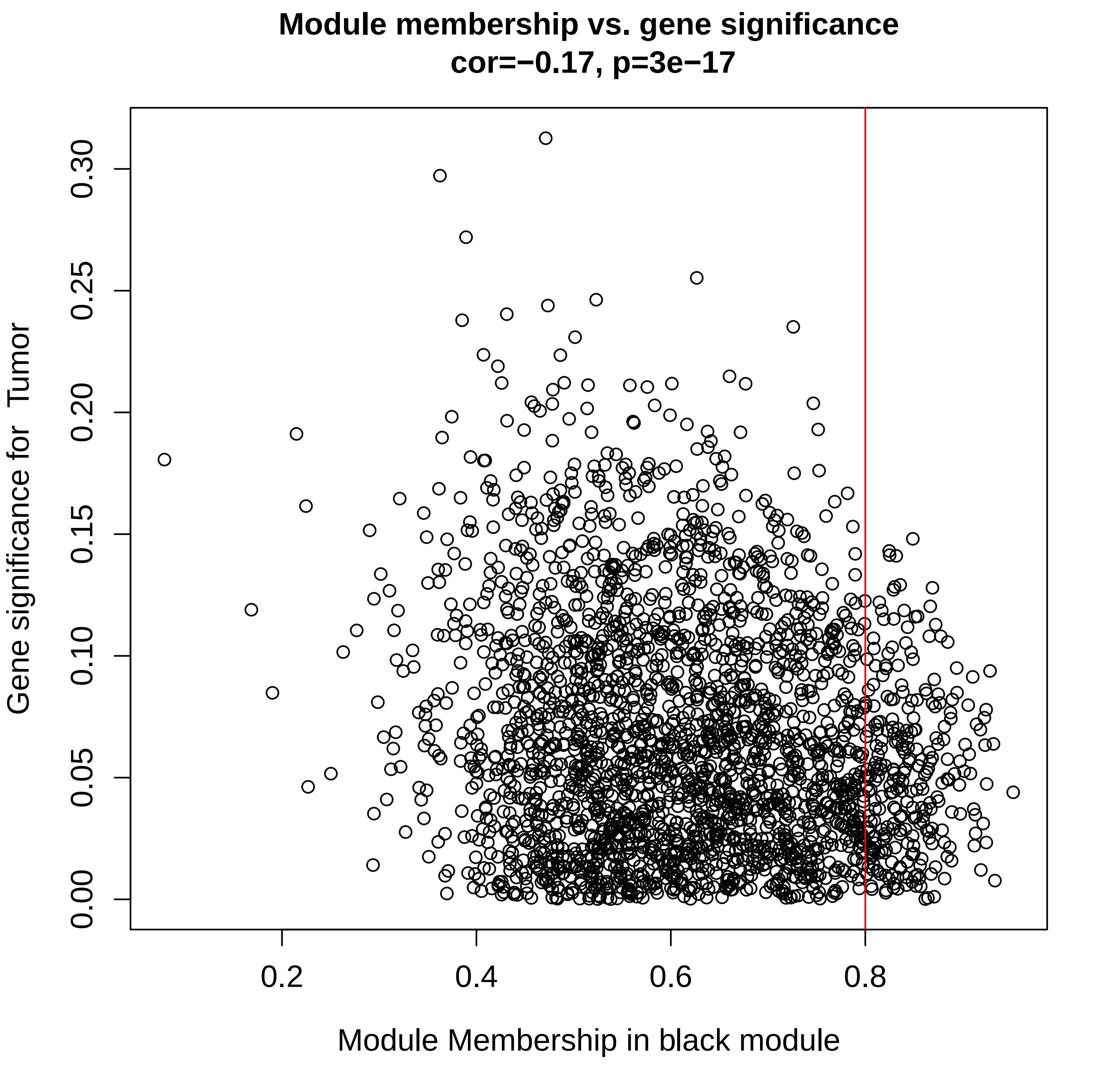

Module membership vs. gene significance
cor=−0.17, p=3e−17
Gene significance for Tumor
0.00
0.05
0.10
0.15
0.20
0.25
0.30
0.2
0.4
0.6
0.8
Module Membership in black module

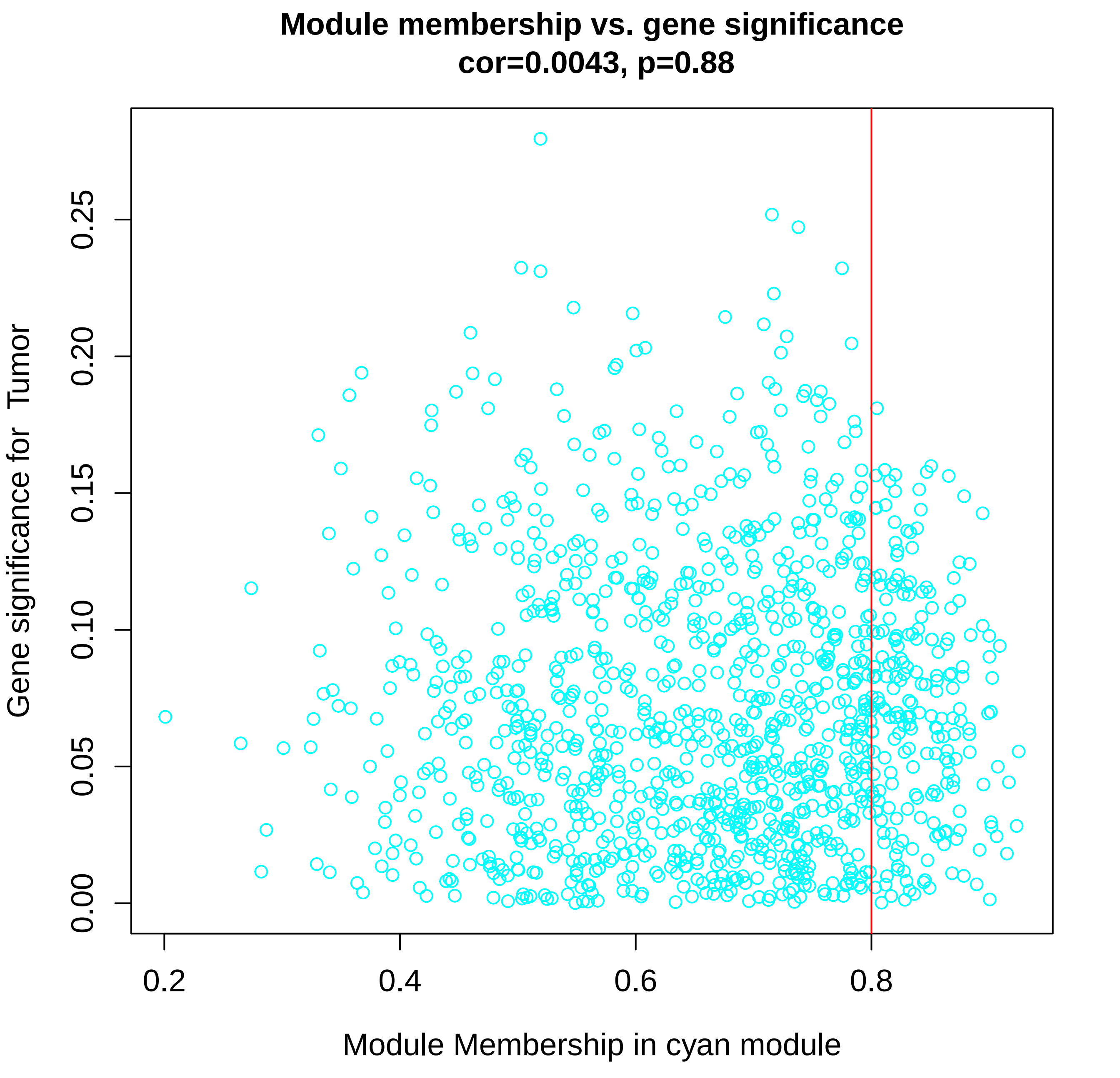

Module membership vs. gene significance
cor=0.0043, p=0.88
Gene significance for Tumor
0.00
0.05
0.10
0.15
0.20
0.25
0.2
0.4
0.6
0.8
Module Membership in cyan module

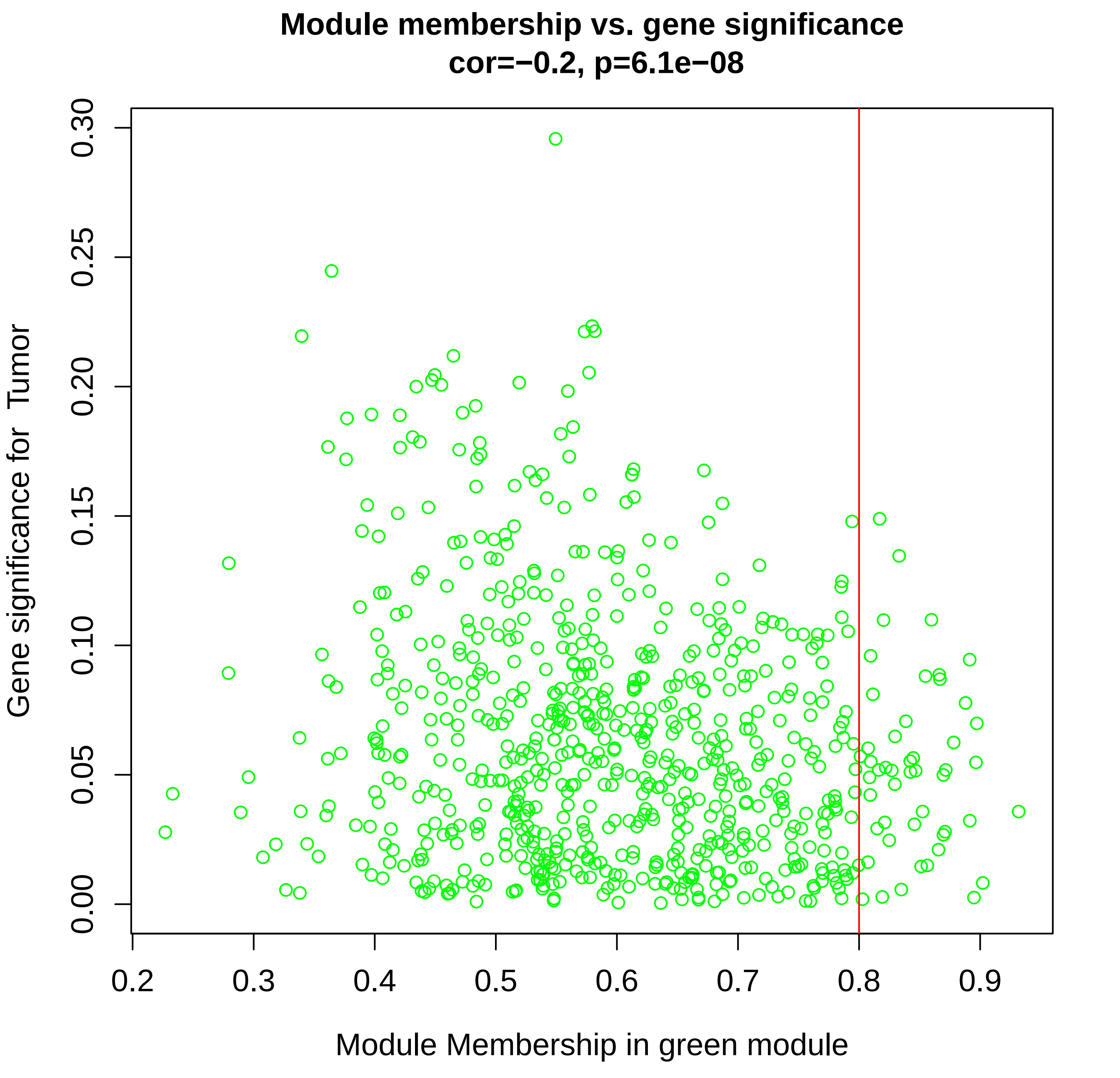
Module membership vs. gene significance
cor=−0.2, p=6.1e−08
Gene significance for Tumor
Module Membership in green module
0.00
0.05
0.10
0.15
0.20
0.25
0.30
0.2
0.3
0.4
0.5
0.6
0.7
0.8
0.9

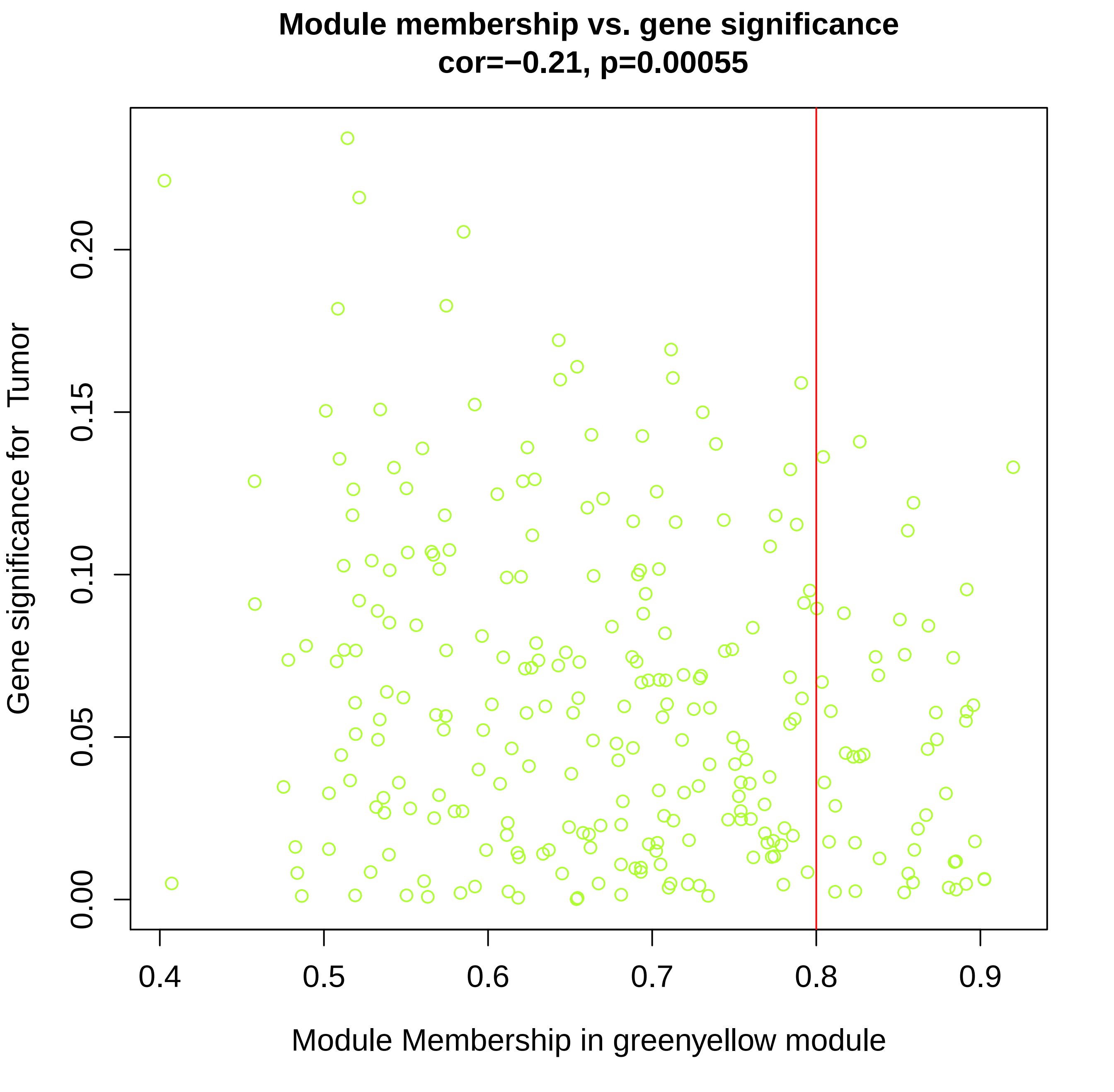
Module membership vs. gene significance
cor=−0.21, p=0.00055
Gene significance for Tumor
0.00
0.05
0.10
0.15
0.20
0.4
0.5
0.6
0.7
0.8
0.9
Module Membership in greenyellow module

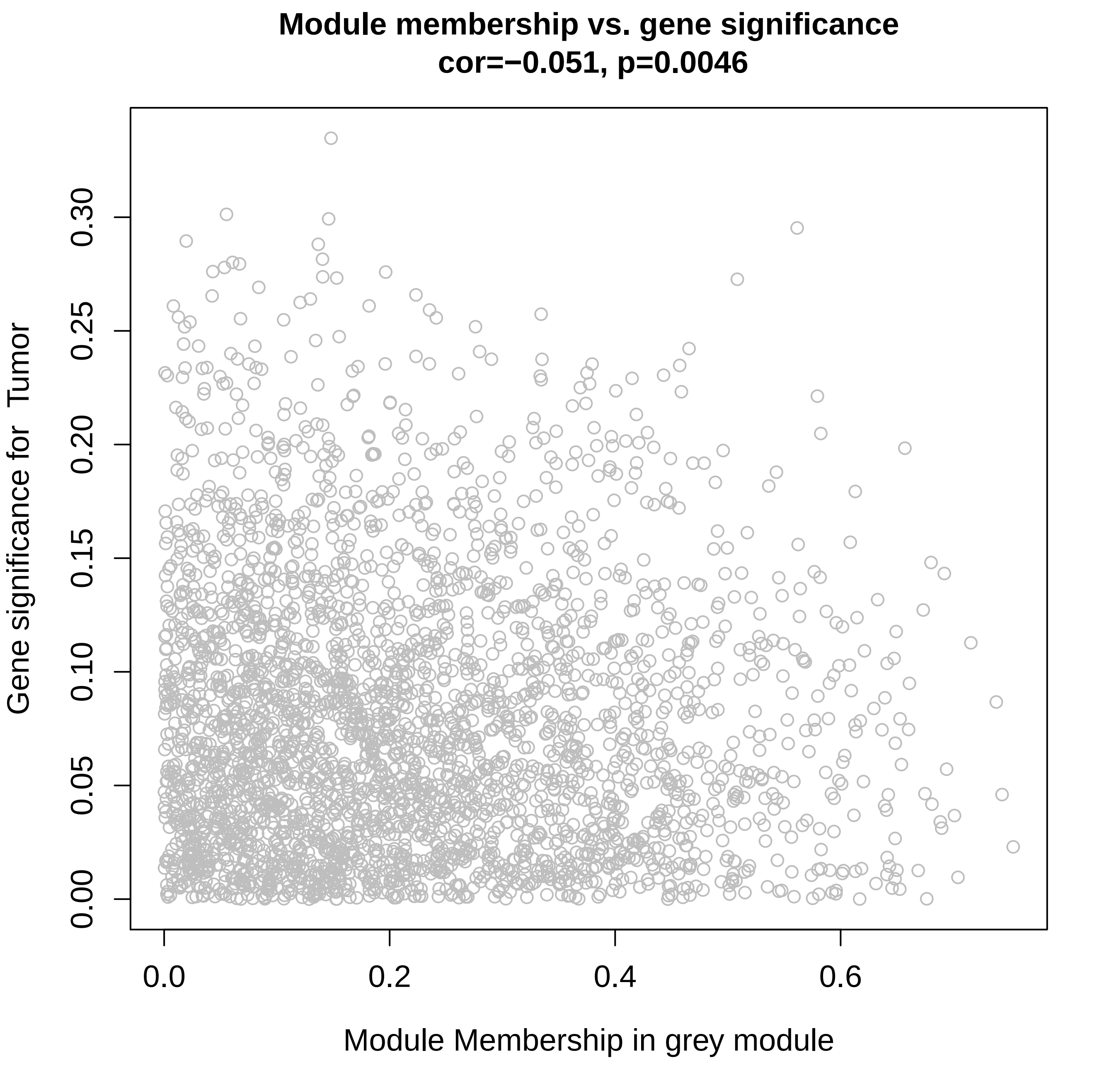
Module membership vs. gene significance
cor=−0.051, p=0.0046
Gene significance for Tumor
0.00
0.05
0.10
0.15
0.20
0.25
0.30
0.0
0.2
0.4
0.6
Module Membership in grey module

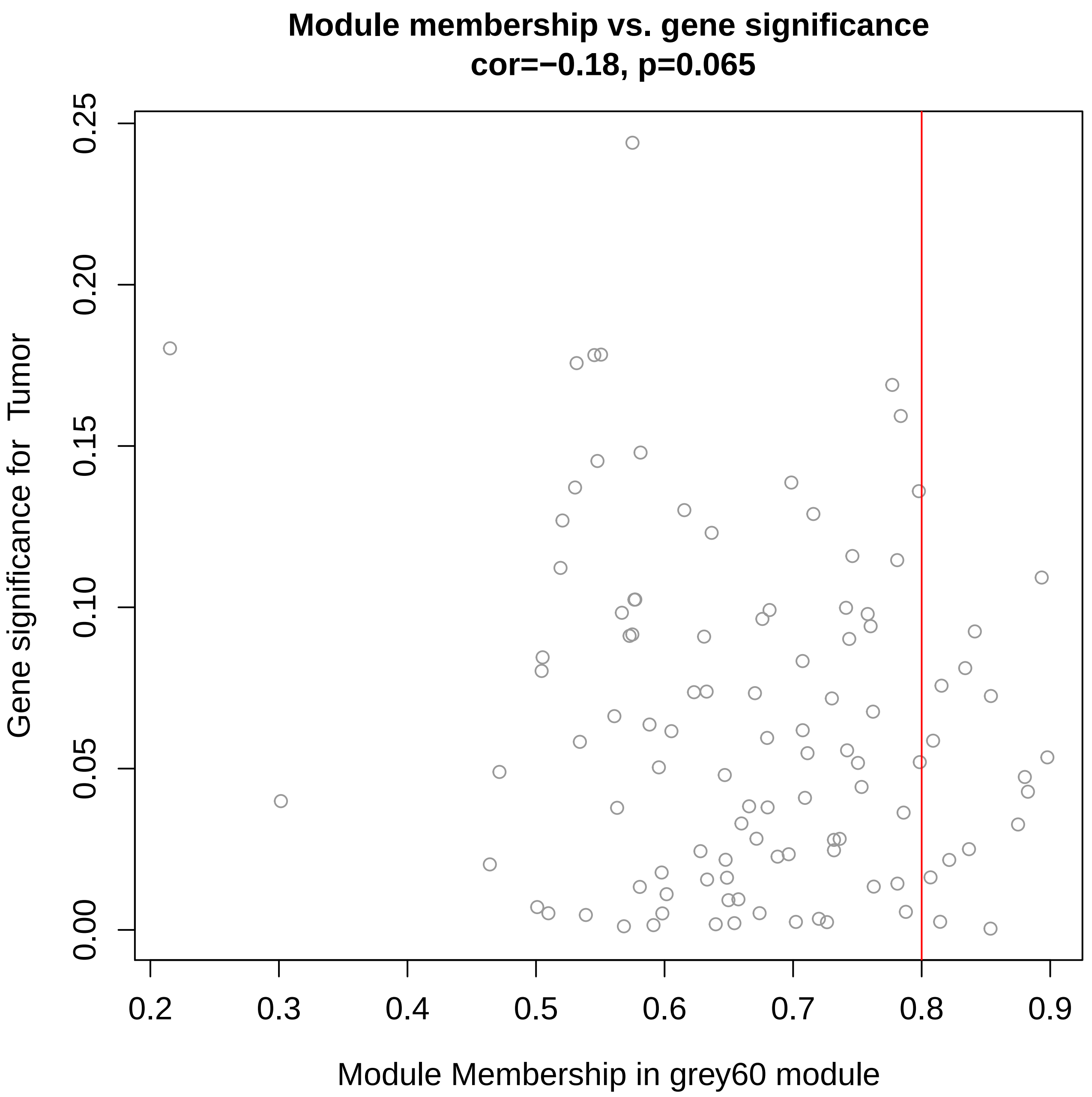
Module membership vs. gene significance
cor=−0.18, p=0.065
Gene significance for Tumor
Module Membership in grey60 module
0.00
0.05
0.10
0.15
0.20
0.25
0.2
0.3
0.4
0.5
0.6
0.7
0.8
0.9

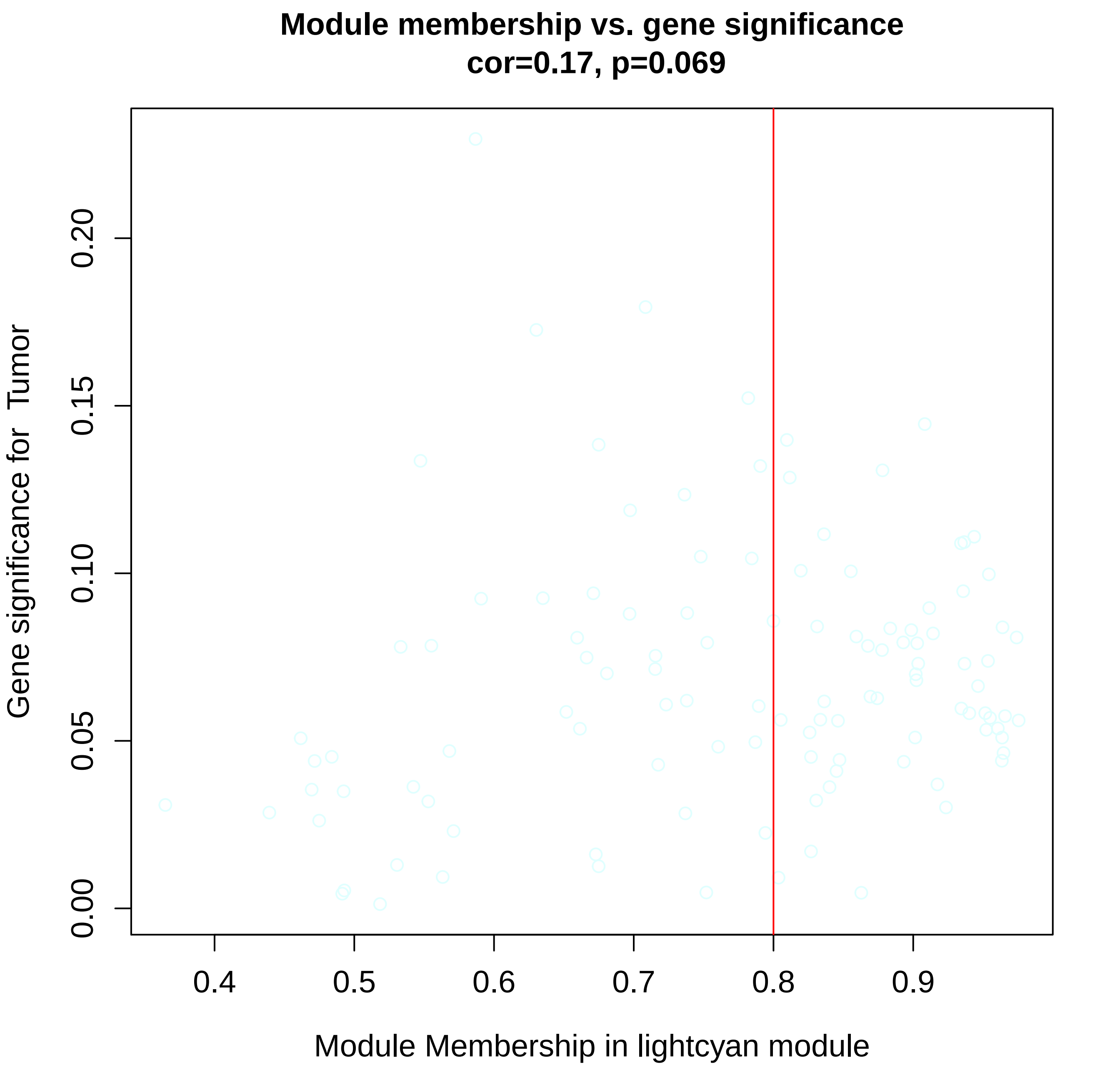
Module membership vs. gene significance
cor=0.17, p=0.069
Gene significance for Tumor
0.00
0.05
0.10
0.15
0.20
0.4
0.5
0.6
0.7
0.8
0.9
Module Membership in lightcyan module

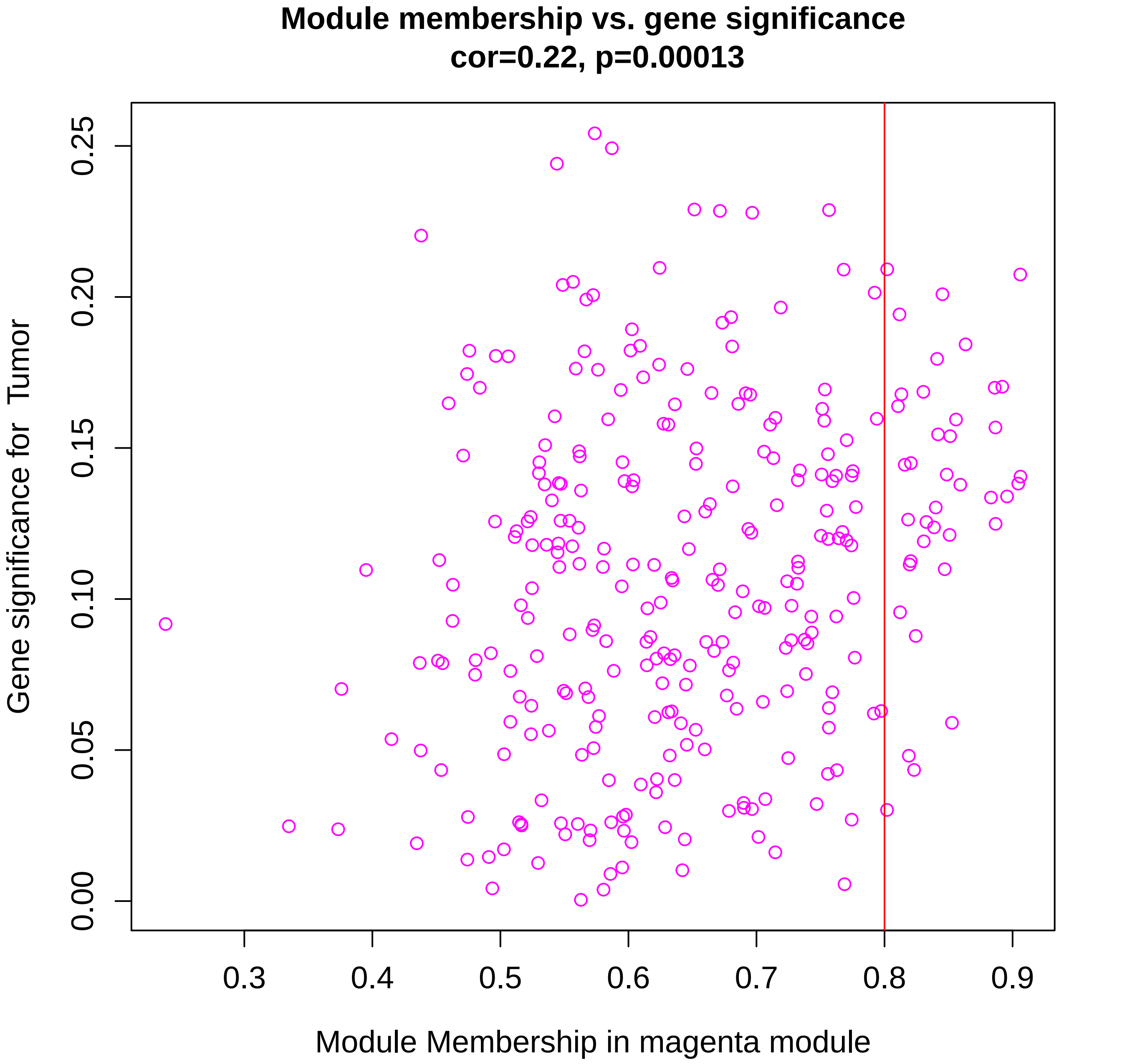

Module membership vs. gene significance
cor=0.22, p=0.00013
Gene significance for Tumor
Module Membership in magenta module
0.00
0.05
0.10
0.15
0.20
0.25
0.3
0.4
0.5
0.6
0.7
0.8
0.9

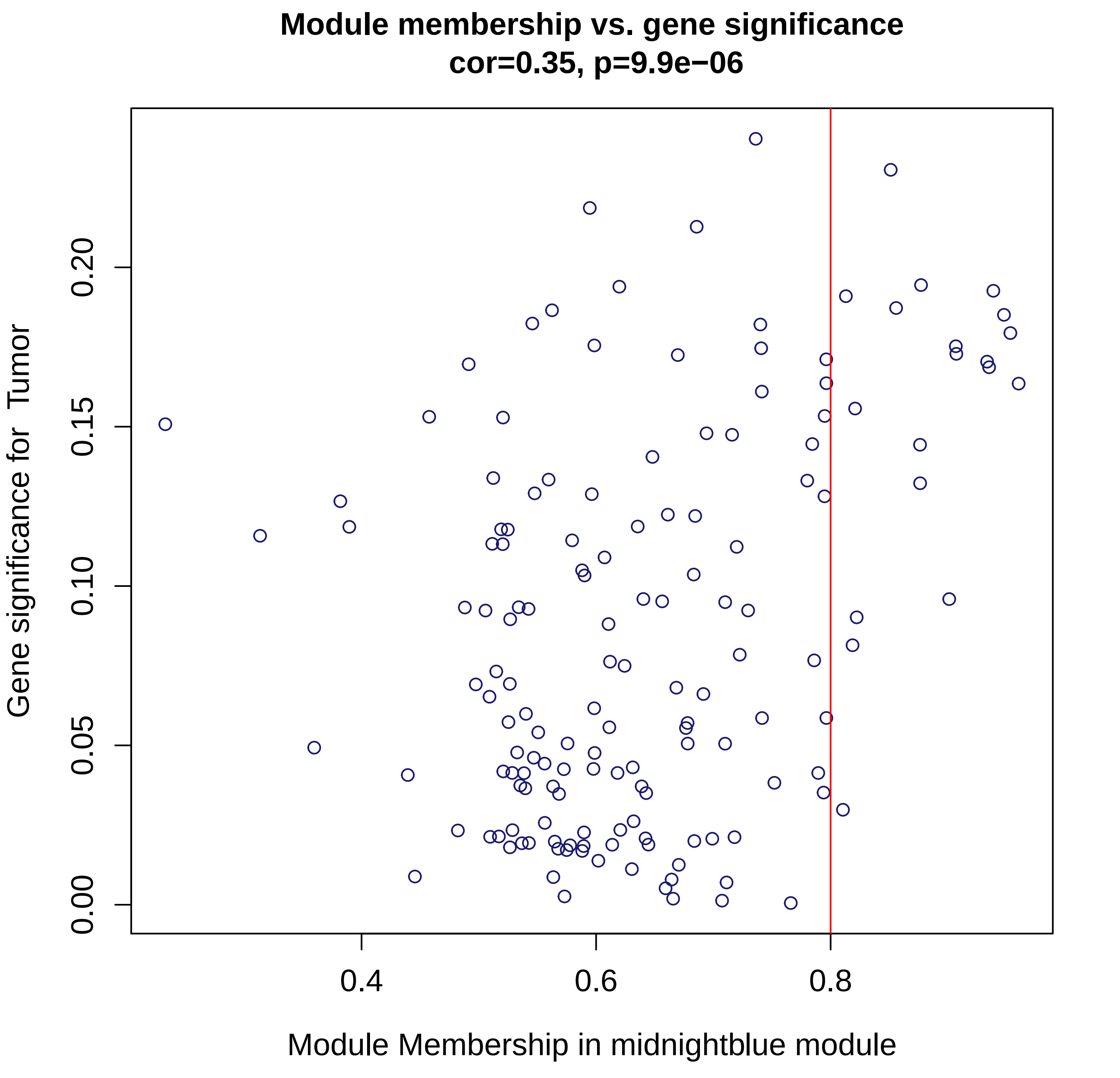
Module membership vs. gene significance
cor=0.35, p=9.9e−06
Gene significance for Tumor
0.00
0.05
0.10
0.15
0.20
0.4
0.6
0.8
Module Membership in midnightblue module

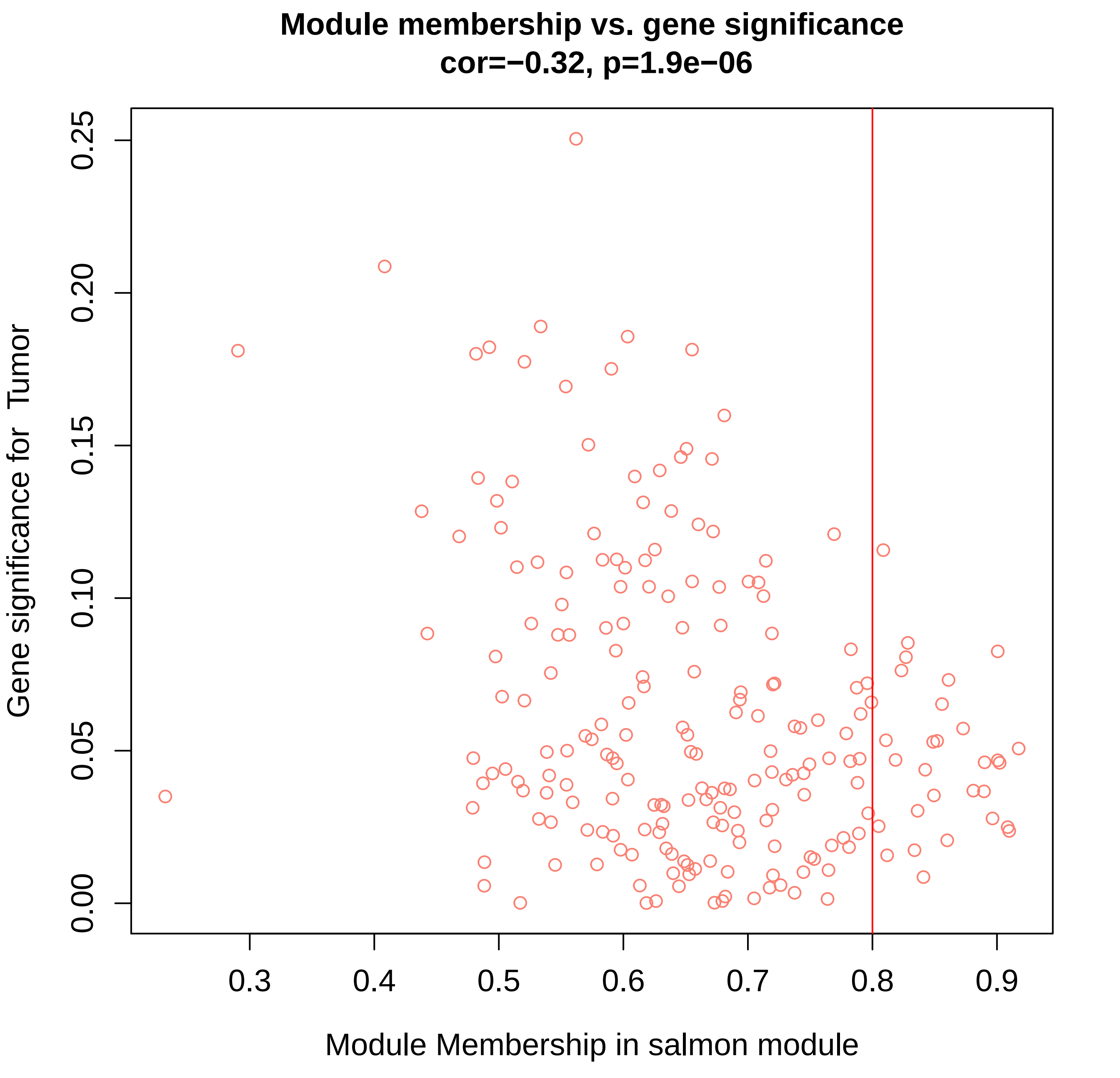

Module membership vs. gene significance
cor=−0.32, p=1.9e−06
Gene significance for Tumor
0.00
0.05
0.10
0.15
0.20
0.25
0.3
0.4
0.5
0.6
0.7
0.8
0.9
Module Membership in salmon module

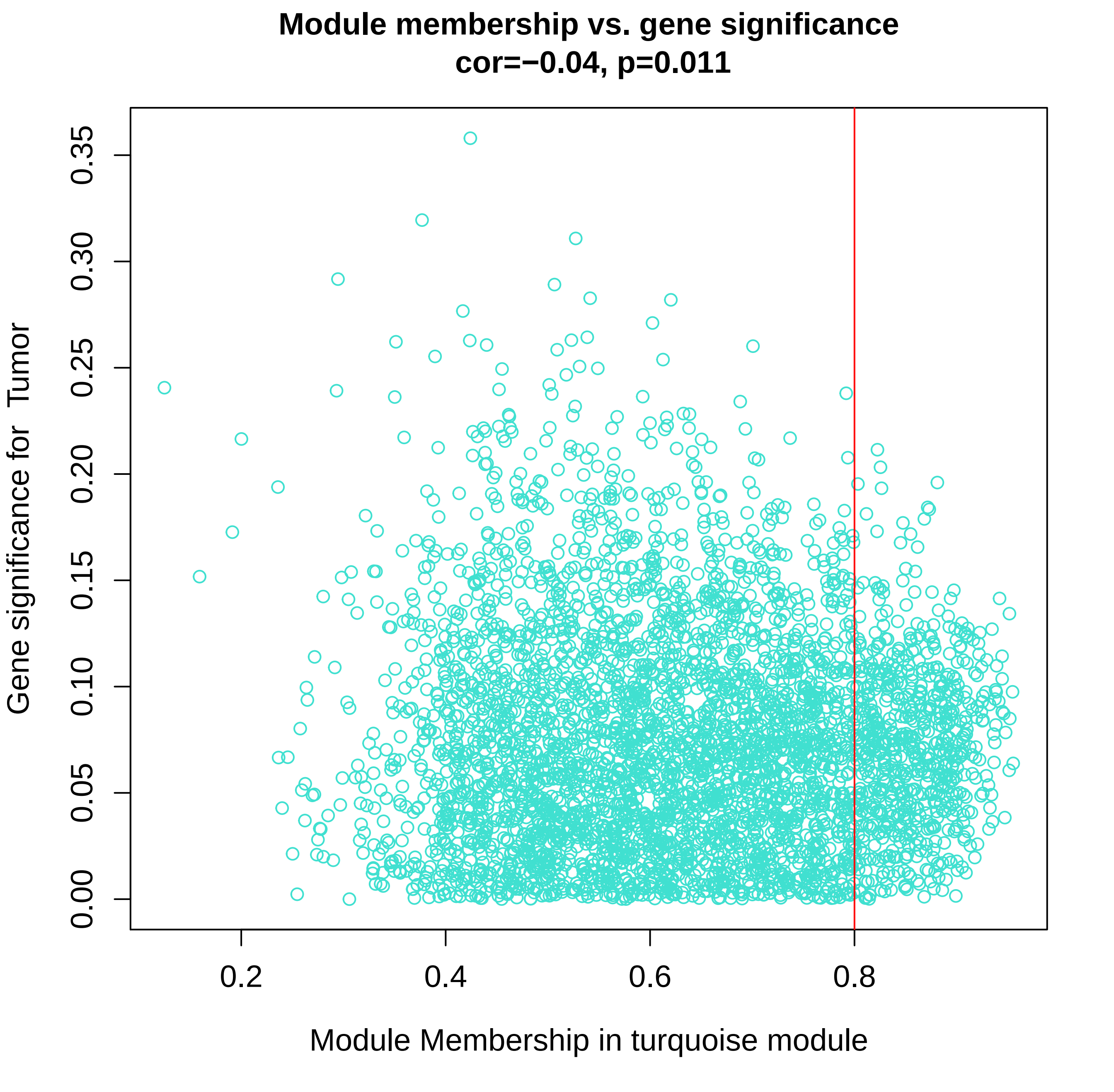

Module membership vs. gene significance
cor=−0.04, p=0.011
Gene significance for Tumor
0.00
0.05
0.10
0.15
0.20
0.25
0.30
0.35
0.2
0.4
0.6
0.8
Module Membership in turquoise module
